# PD-1/PD-L1 inhibitors plus chemotherapy versus chemotherapy alone for Asian patients with advanced triple-negative breast cancer: a phase III RCTs based meta-analysis

**DOI:** 10.3389/fonc.2025.1540538

**Published:** 2025-02-28

**Authors:** Hua Ruan, Yubin Zou, Lifeng Huang, Wenjuan Zha, Qingqing Ouyang, Ling Yang

**Affiliations:** ^1^ Department of Oncology, Xinyu People’s Hospital, Xinyu, China; ^2^ Department of Oncology, Fenyi People’s Hospital, Xinyu, China

**Keywords:** PD-1/PD-L1 inhibitors, chemotherapy, triple-negative breast cancer, randomized controlled trials, meta-analysis

## Abstract

**Background:**

Advanced triple-negative breast cancer (TNBC) presents significant therapeutic challenges, particularly in Asian populations, which exhibit distinct biological and genetic characteristics. Immunotherapy combined with chemotherapy has emerged as a promising approach; however, its efficacy compared to chemotherapy alone remains under investigation. This meta-analysis aims to evaluate the clinical outcomes of PD-1/PD-L1 inhibitors combined with chemotherapy (PIC) versus chemotherapy alone in the treatment of advanced TNBC in Asian patients.

**Methods:**

A systematic literature search was performed across six databases for phase 3 randomized controlled trials (RCTs). Only studies comparing the outcomes of PIC versus chemotherapy alone in patients with advanced TNBC, including subgroup analyses of Asian populations, were included. Data were pooled to assess overall survival (OS), progression-free survival (PFS), responses, and safety profiles.

**Results:**

A total of 1041 patients from five phase 3 RCTs were included in the final analysis. Compared to chemotherapy alone, PIC therapy significantly improved PFS (hazard ratio [HR]: 0.74 [0.62, 0.88], P = 0.0008). No significant difference was observed in OS (HR: 0.78 [0.55, 1.12], P = 0.18), although a slight trend favoring PIC therapy was noted. Among PD-L1-positive patients, both OS (HR: 0.62 [0.44, 0.86], P = 0.005) and PFS (HR: 0.66 [0.50, 0.86], P = 0.003) were significantly improved in the PIC group. The PIC group also exhibited a substantially higher OS rate at 12–36 months and a higher PFS rate at 6–30 months. However, the incidence of immune-related AEs (irAEs) (risk ratio [RR]: 1.69 [1.33, 2.15], P < 0.0001) and grade 3–5 irAEs (RR: 3.11 [1.59, 6.10], P = 0.001) was significantly higher in the PIC group. The most common irAEs in the PIC group were hypothyroidism (14.40%), dermatitis (10.00%), and infusion reactions (8.85%). Both treatment groups exhibited similar response rates and treatment-related AEs (TRAEs).

**Conclusions:**

In Asian patients with advanced TNBC, PIC significantly improved survival compared to chemotherapy alone. Although the combination therapy was associated with a higher incidence of irAEs, its clinical benefits support its use as a viable treatment option for this population.

**Systematic review registration:**

https://www.crd.york.ac.uk/prospero/, identifier CRD42024622428.

## Introduction

Triple-negative breast cancer (TNBC) accounts for approximately 15-20% of all breast cancer cases and is associated with a poorer prognosis (1, 2). Chemotherapy has long been the cornerstone of treatment for advanced TNBC, offering some degree of efficacy. However, the prognosis remains unsatisfactory, with limited treatment options available (3). In recent years, the advent of immune checkpoint inhibitors, particularly PD-1/PD-L1 inhibitors, has revolutionized cancer therapy, showing promising results in various malignancies, including TNBC (4).

Several randomized controlled trials (RCTs) have investigated the impact of PD-1/PD-L1 inhibitors plus chemotherapy (PIC) in TNBC patients (5–9). For instance, the IMpassion130 trial demonstrated that the addition of atezolizumab to nab-paclitaxel resulted in a significant improvement in progression-free survival (PFS) and overall survival (OS) for patients with PD-L1-positive metastatic TNBC (5). Similarly, the KEYNOTE-355 trial showed that pembrolizumab, a PD-1 inhibitor, combined with chemotherapy, led to significant improvements in both PFS and OS in PD-L1-positive metastatic TNBC patients (8). A comprehensive meta-analysis by Wang et al. evaluated the efficacy and safety of PIC for patients with unresectable TNBC. The study concluded that this combination therapy significantly improved PFS and OS compared to chemotherapy alone, particularly in PD-L1-positive populations (10). Despite these encouraging outcomes, the applicability of these findings to Asian populations remains uncertain. TNBC exhibits distinct biological and genetic characteristics across different ethnic groups, which can influence treatment responses (11). Moreover, the prevalence of TNBC subtypes and PD-L1 expression levels may vary among populations, potentially affecting the efficacy of immunotherapy. Notably, Asian patients have been underrepresented in major clinical trials, resulting in a paucity of evidence regarding the effectiveness of these therapies in this demographic (12).

Given these considerations, our meta-analysis seeks to compare the PIC versus chemotherapy alone focusing specifically on Asian patients with advanced TNBC. By synthesizing data from phase III RCTs, we aim to provide robust evidence to guide clinical decision-making and optimize treatment strategies for this population.

## Materials and methods

### Search strategy

The search strategy employed the following keywords: “PD-1/PD-L1 inhibitors”, “Breast Cancer”, and “Randomized”. A comprehensive search was conducted across six databases (PubMed, ScienceDirect, Cochrane Library, Scopus, EMBASE, and Web of Science) from their inception up to November 12, 2024 (**Supplementary Table S1**).

### Selection criteria

Inclusion criteria (PICOS) were as follows: (1) Participants: Asian patients with advanced TNBC; (2) Intervention and Control: PIC versus chemotherapy alone; (3) Outcomes: OS, PFS, responses, and adverse events (AEs); (4) Study design: Phase III RCTs. Animal studies, reviews, meta-analyses, and case reports were excluded.

### Data extraction

Two researchers independently extracted information on study characteristics (e.g., PD-1/PD-L1 type, period), patient characteristics (e.g., age, metastatic disease), survival metrics (e.g., OS, PFS), response rates (e.g., objective response rate [ORR]), and AEs (e.g., treatment-related AEs [TRAEs]). Missing data were requested from the corresponding authors, and any discrepancies were resolved through re-assessment.

### Quality assessment

The Cochrane Risk Assessment Tool and the Jadad scale, a 5-point system where scores of 3 to 5 indicate high quality, were used to evaluate the quality of the included studies (13, 14). The GRADE approach was employed to assess results, classifying them into four categories: high, medium, low, and very low (15).

### Statistical analysis

Data analysis was performed using Review Manager 5.3 and STATA 12.0. Hazard ratios (HR) were used for survival outcomes, while risk ratios (RR) were employed for dichotomous variables. Survival rates for OS (OSR) and PFS (PFSR) were evaluated over periods ranging from 3 to 36 months. For low heterogeneity (*I*² < 50% or P > 0.1), a fixed-effects model was utilized, while a random-effects model was employed for greater heterogeneity. Statistical significance was set at P < 0.05. Publication bias was assessed visually through funnel plots. The study followed PRISMA guidelines and was registered with PROSPERO (ID: CRD42024622428).

## Results

### Search results

**Figure 1:**
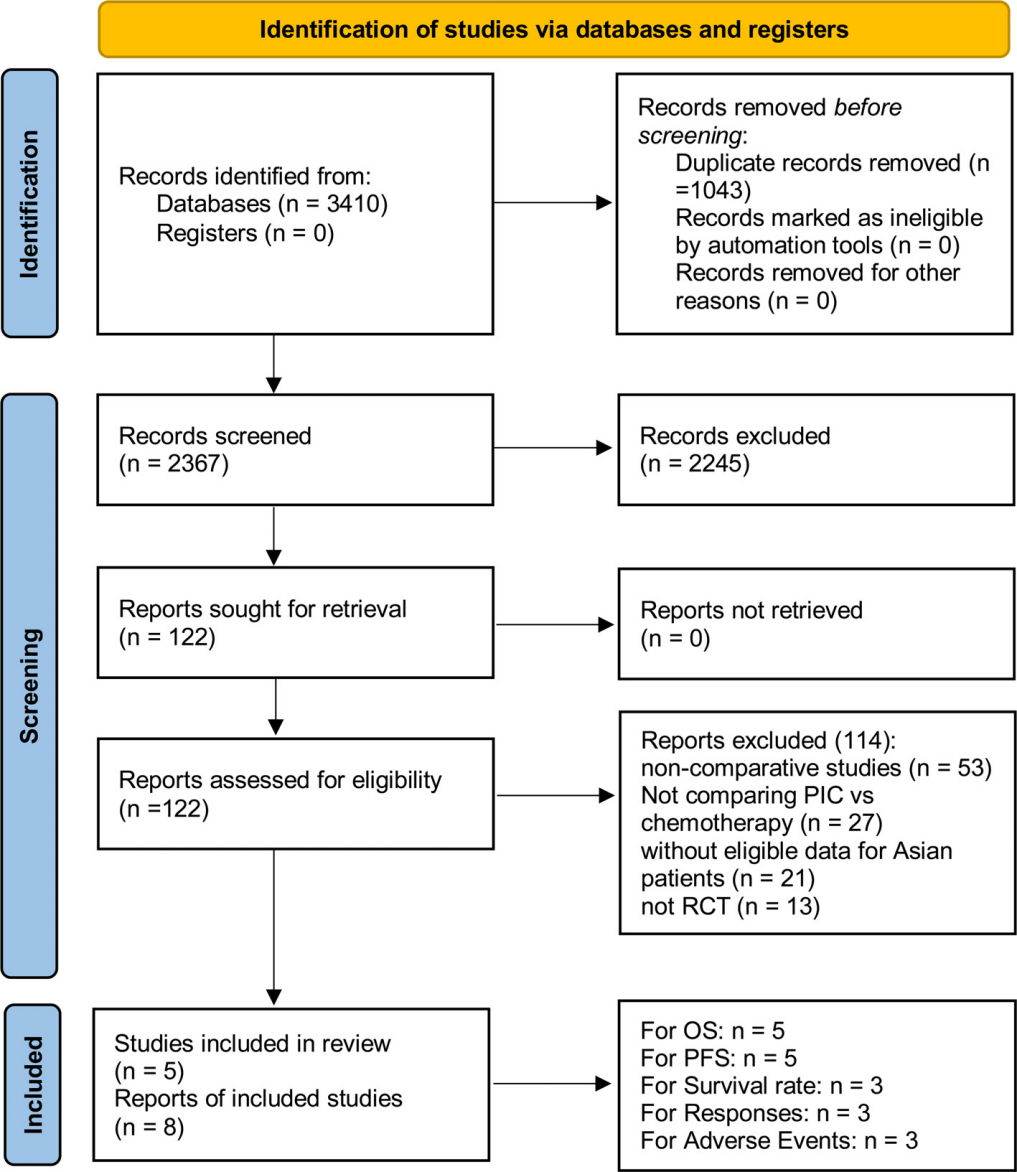
Flow chart.

From 2367 screened studies, 8 studies derived from 5 phase III RCTs (IMpassion130, IMpassion131, IMpassion132, KEYNOTE-355, and TORCHLIGHT), encompassing 1041 Asian patients with TNBC, were included (**Figure 1**) (5–9, 16–18). The baseline characteristics of these RCTs are detailed in **Table 1**. IMpassion130, IMpassion131, IMpassion132, and KEYNOTE-355 are international multicenter trials, whereas TORCHLIGHT is a multicenter trial conducted in China. In these four international multicenter studies, IMpassion130 and KEYNOTE-355 provide detailed data analysis of the Asian population, while IMpassion131 and IMpassion132 only include subgroup analyses of survival data for the Asian population. All studies were evaluated as high quality (**Supplementary Figure S1**, **Supplementary Table S2**). Using the GRADE approach, the quality of the results was classified as medium to high (**Supplementary Table S3**).

**Table 1:** Baseline characteristics of the included studies.

Study	Country	Groups	Patients	Age (Mean, year)	ECOG PS		Metastatic disease	Endpoints	PD-1/PD-L1 type	Chemotherapy type	Follow up (months)
					0	1					
**IMpassion130 (NCT02425891, 2016.08-2017.05)**											
Emens 2021 (5), Iwata 2019 (16), Schmid 2018 (17)	Global multicenter a	PIC	34	55	28	6	32	AEs, Responses, OS, PFS	Atezolizumab	Nab-paclitaxel	18.8
		Chemotherapy	31	54	27	4	22				
**IMpassion131 (NCT03125902, 2017.08-2019.09)**											
Miles 2021 (6)^a^	Global multicenter	PIC	123	–	–	–	–	OS, PFS	Atezolizumab	Paclitaxel	14.2
		Chemotherapy	66	–	–	–	–				
**IMpassion132 (NCT03371017, 2018.01-2023.08)**											
Dent 2024 (7)^a^	Global multicenter	PIC	48	–	–	–	–	PFS	Atezolizumab	Gemcitabine+carboplatin or Capecitabine	9.8
		Chemotherapy	48	–	–	–	–				
**KEYNOTE-355 (NCT02819518, 2017.01-2018.03)**											
Im 2024 (8), Hattori (18)	Global multicenter	PIC	113	55	79	34	110	AEs, Responses, OS, PFS	Pembrolizumab	Nab-paclitaxel or Paclitaxel or Gemcitabine+carboplatin	43.8
		Chemotherapy	47	50	36	11	46				
**TORCHLIGHT (NCT03777579, 2018.12-2022.11)**											
Jiang 2024 (9)	China multicenter	PIC	353	53	171	183	353	AEs, Responses, OS, PFS	Toripalimab	Nab-paclitaxel	14
		Chemotherapy	178	54	91	87	178				

AE, Adverse event; ECOG PS, Eastern Cooperative Oncology Group performance status; OS, Overall survival; PD-1, Programmed death-1; PD-L1, Programmed death-ligand 1; PFS, Progression-free survival; PIC, PD-1/PD-L1 inhibitors plus chemotherapy.

a IMpassion131 and IMpassion132 only have subgroup analysis of survival data for the Asian population.

### Survival

**Figure 2:**
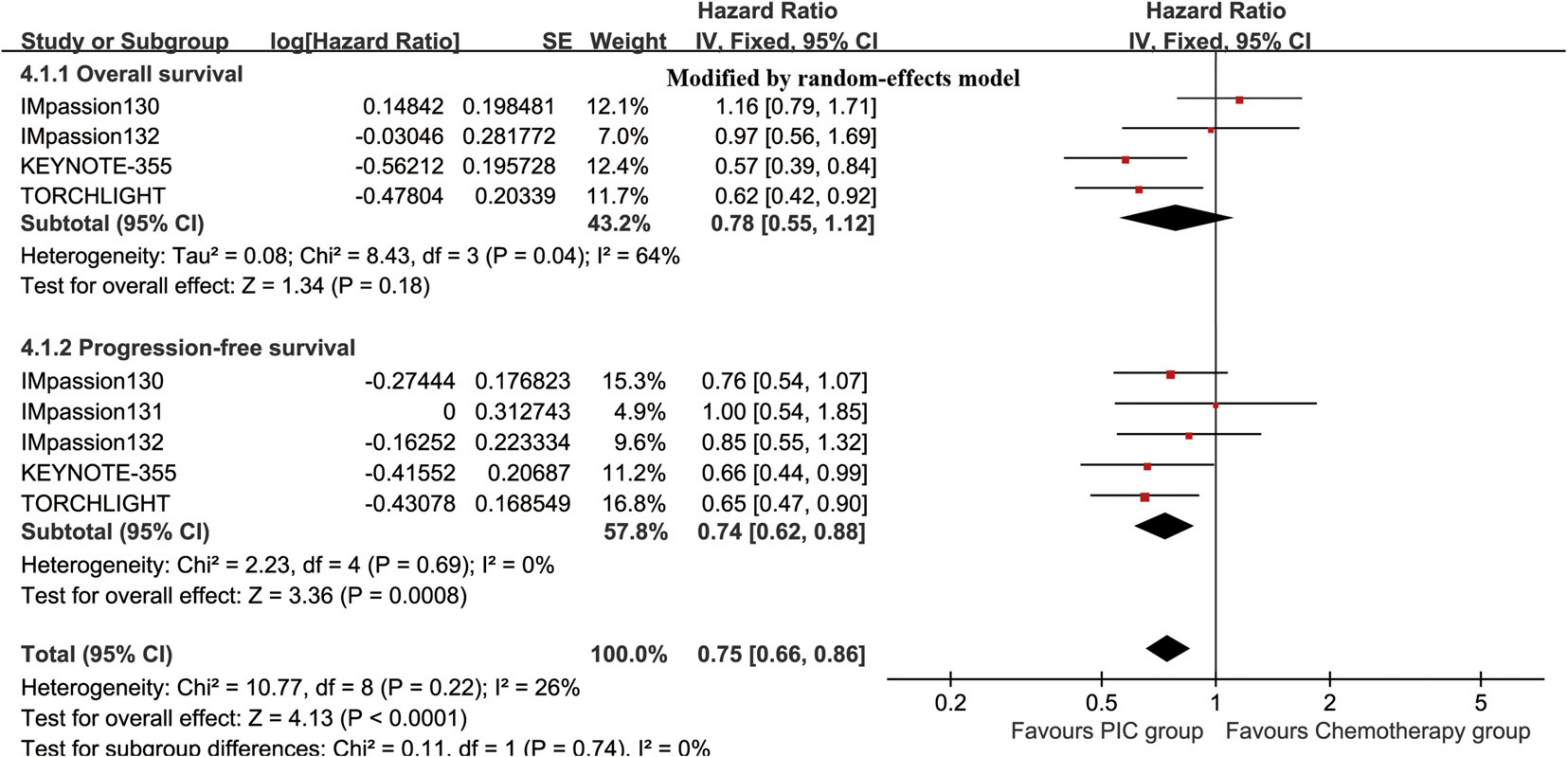
Forest plots of overall survival and progression-free survival associated with PIC versus chemotherapy.

**Figure 3:**
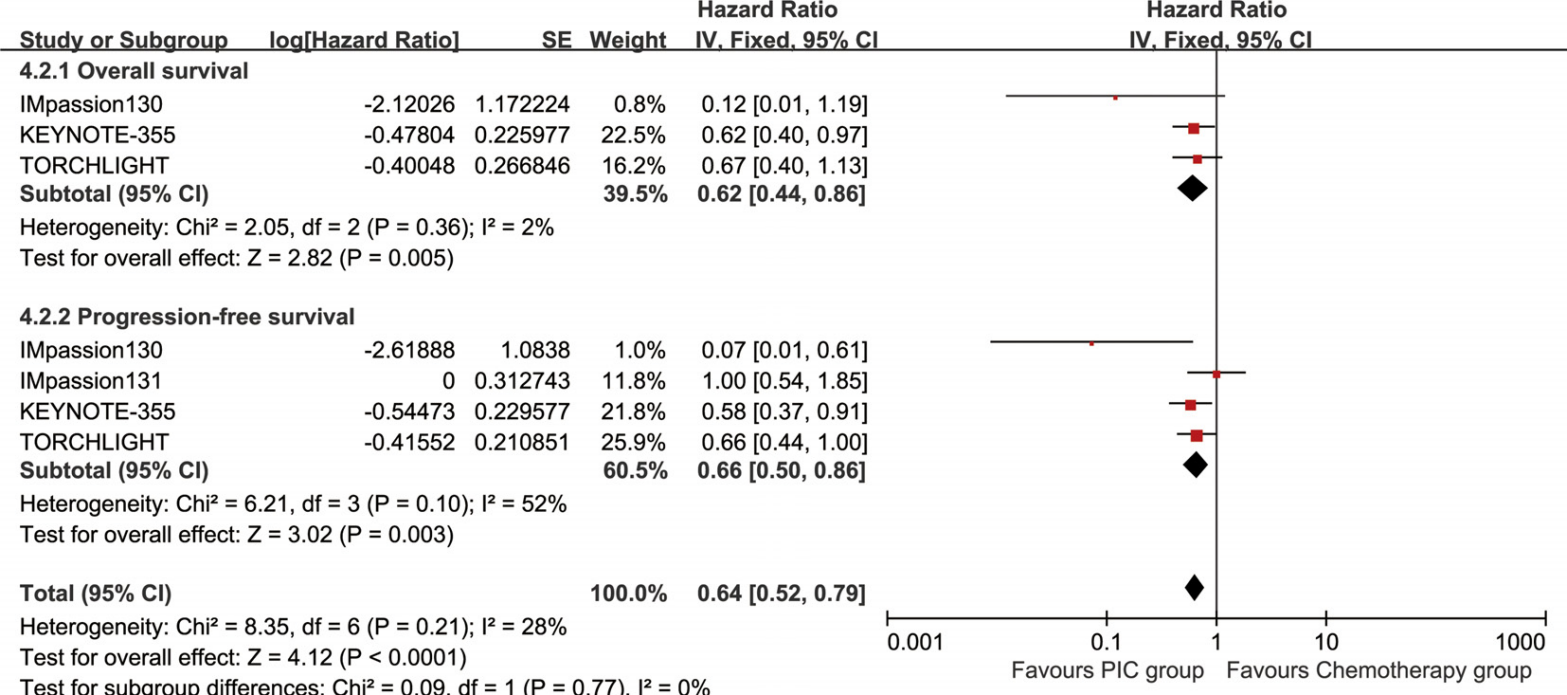
Forest plots of overall survival and progression-free survival associated with PIC versus chemotherapy in PD-L1-positive population.

**Figure 4:**
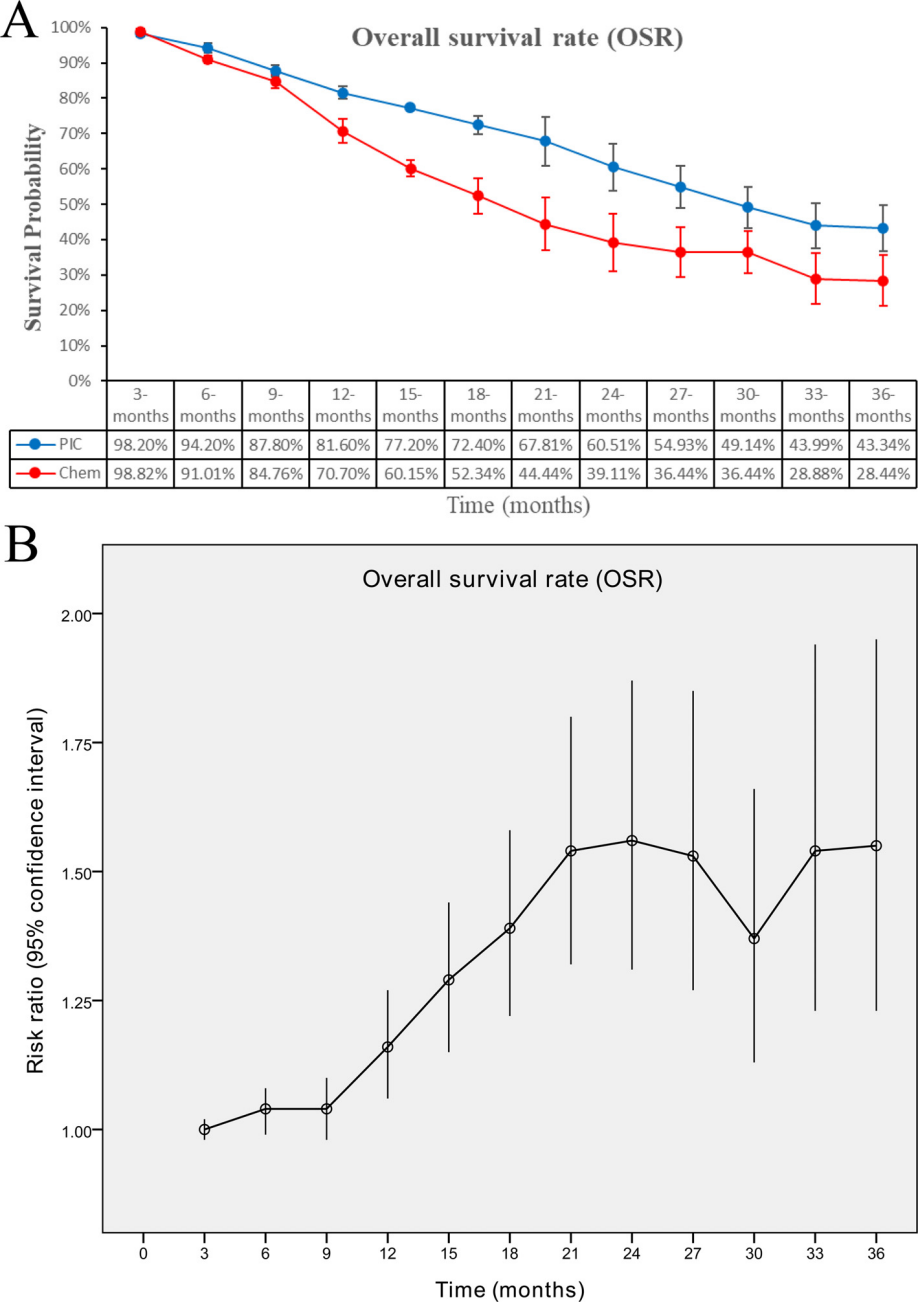
Comparisons of overall survival rate associated with PIC versus chemotherapy. **(A)** OSR at 3-36 months between the two groups; **(B)** trend of risk ratios in OSR.

The OS (HR: 0.78 [0.55, 1.12], P = 0.18) tended to favor the PIC therapy, but no significant difference was observed (**Figure 2**). In the PD-L1-positive subgroup, the PIC group demonstrated better OS (HR: 0.62 [0.44, 0.86], P = 0.005) (**Figure 3**). OSR was significantly higher in the PIC group across 12-36 months (**Figure 4**, **Supplementary Figure S2**).

**Figure 5:**
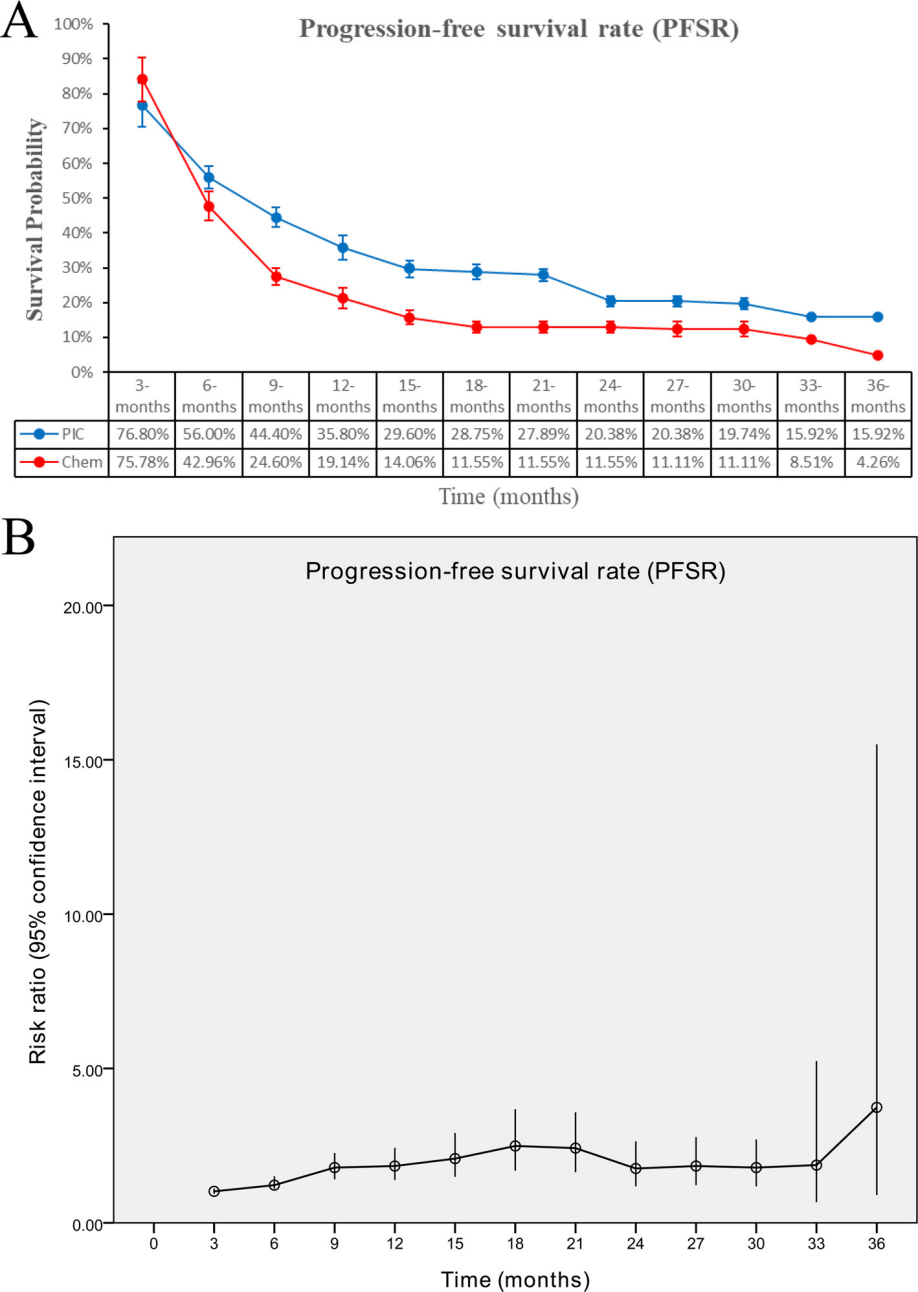
Comparisons of progression-free survival rate associated with PIC versus chemotherapy. **(A)** PFSR at 3-36 months between the two groups; **(B)** trend of risk ratios in PFSR.

The PIC group demonstrated improved progression-free survival (PFS) (HR: 0.74 [0.62, 0.88], P = 0.0008), with a more pronounced effect in the PD-L1-positive subgroup (HR: 0.66 [0.50, 0.86], P = 0.003) (**Figure 2**, **3**). PFSR was significantly higher in the PIC group across 6-30 months (**Figure 5**, **Supplementary Figure S3**).

### Responses

**Figure 6:**
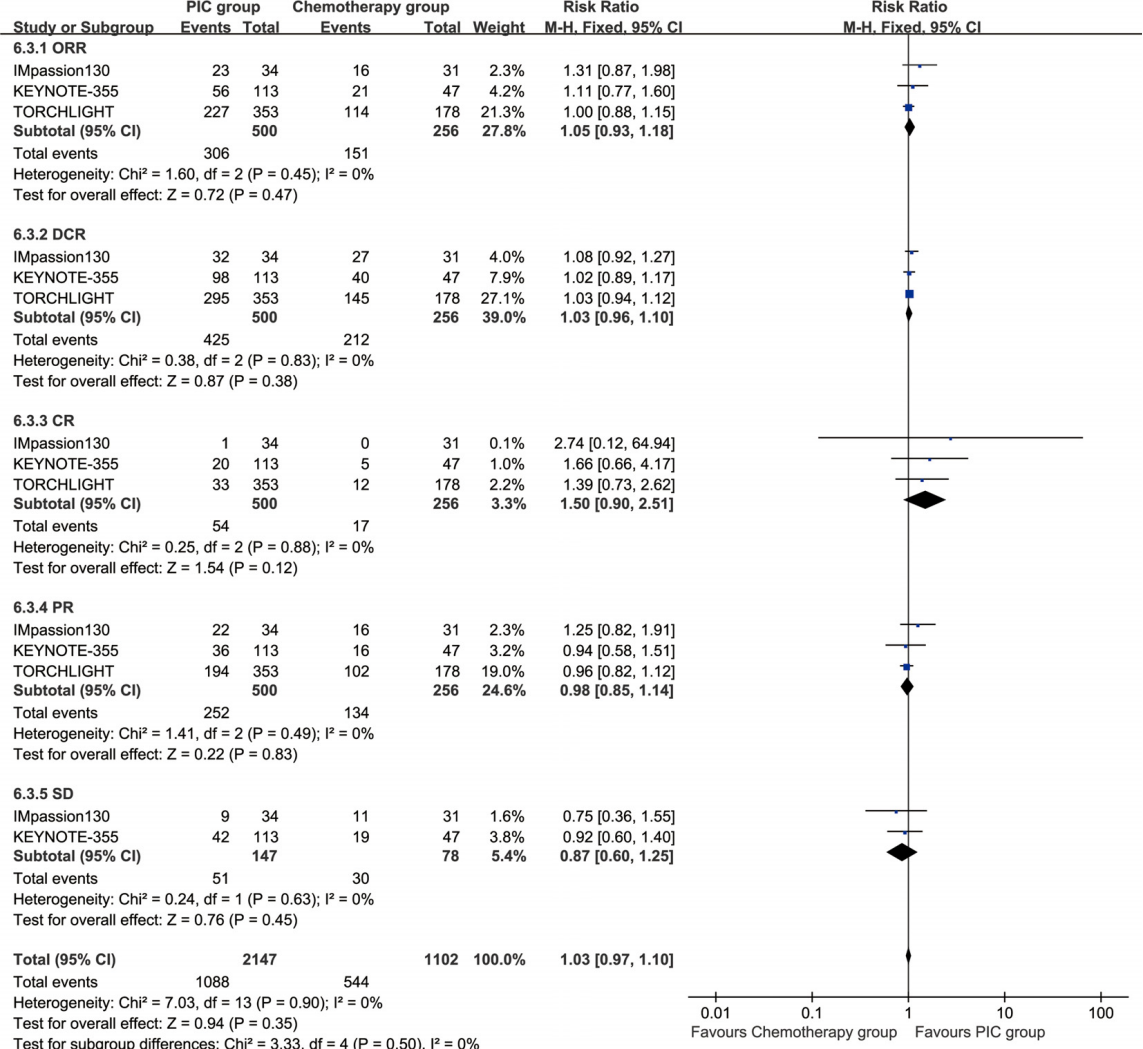
Forest plots of responses associated with PIC versus chemotherapy.

The ORR (RR: 1.05 [0.93, 1.18], P = 0.47), DCR (RR: 1.03 [0.96, 1.10], P = 0.38), complete response (CR) (RR: 1.50 [0.90, 2.51], P = 0.12), partial response (PR) (RR: 0.98 [0.85, 1.14], P = 0.83), and stable disease (SD) (RR: 0.87 [0.60, 1.25], P = 0.45) were comparable between the two groups (**Figure 6**).

### Safety

In summary, the PIC group experienced higher rates of immune-related AEs (irAEs) (RR: 1.69 [1.33, 2.15], P < 0.0001), grade 3-5 irAEs (RR: 3.11 [1.59, 6.10], P = 0.001), TRAEs-related deaths (RR: 1.57 [1.13, 2.19], P = 0.007), TRAEs leading to discontinuation (RR: 2.43 [1.32, 4.45], P = 0.004), and TRAEs leading to interruption (RR: 1.44 [1.19, 1.75], P = 0.0002). Total TRAEs and grade 3-5 TRAEs were comparable between the groups (**Table 2**, **Supplementary Figure S4**).

**Table 2:** Summary of adverse events.

Adverse events	PIC		Chemotherapy		Risk ratio [95% CI]	P
	Event/total	%	Event/total	%		
TRAEs	494/500	98.80%	249/256	97.27%	1.02 [0.99, 1.04]	0.17
Grade 3-5 TRAEs	298/500	59.60%	143/256	55.86%	1.03 [0.91, 1.17]	0.64
TRAEs-related deaths	122/500	24.40%	39/256	15.23%	1.57 [1.13, 2.19]	0.007
TRAEs leading to discontinuation	58/500	11.60%	11/256	4.30%	2.43 [1.32, 4.45]	0.004
TRAEs leading to interruption	237/500	47.40%	85/256	33.20%	1.44 [1.19, 1.75]	0.0002
irAEs	201/500	40.20%	64/256	25.00%	1.69 [1.33, 2.15]	< 0.0001
Grade 3-5 irAEs	58/500	11.60%	9/256	3.52%	3.11 [1.59, 6.10]	0.001

AE, Adverse event; CI, Confidence interval; irAE, Immune-related adverse event; PD-1, Programmed death-1; PD-L1, Programmed death-ligand 1; PIC, PD-1/PD-L1 inhibitors plus chemotherapy; RR, Risk ratio; TRAE, Treatment-related adverse event.

In the analysis of TRAEs, the PIC group experienced higher rates of any grade nasopharyngitis, nausea, asthenia, stomatitis, hypoesthesia, vomiting, hypothyroidism, and grade 3-5 AST increased (**Tables 3**, **4**, **S4**, **S5**).

**Table 3:** Any grade treatment-related adverse events (> 20% in the PIC group).

TRAEs	PIC		Chemotherapy		Risk ratio [95% CI]	P
	Event/total	%	Event/total	%		
Leukopenia	257/353	72.80%	134/178	75.28%	0.97 [0.87, 1.07]	0.53
Alopecia	315/500	63.00%	154/256	60.16%	1.08 [0.96, 1.22]	0.18
Anaemia	280/500	56.00%	132/256	51.56%	1.05 [0.92, 1.21]	0.48
Neutrophil count decreased	80/147	54.42%	39/78	50.00%	1.02 [0.79, 1.32]	0.88
Neutropenia	266/500	53.20%	123/256	48.05%	1.08 [0.95, 1.23]	0.26
White blood cell count decreased	67/147	45.58%	30/78	38.46%	1.44 [0.41, 5.02]	0.57
AST increased	165/387	42.64%	92/209	44.02%	0.92 [0.77, 1.11]	0.41
ALT increased	163/387	42.12%	93/209	44.50%	0.91 [0.75, 1.10]	0.34
Nasopharyngitis	11/34	32.35%	3/31	9.68%	3.34 [1.03, 10.88]	0.05
Nausea	161/500	32.20%	60/256	23.44%	1.39 [1.08, 1.78]	0.01
Asthenia	111/353	31.44%	39/178	21.91%	1.44 [1.05, 1.97]	0.03
Decreased platelet count	31/113	27.43%	11/47	23.40%	1.17 [0.64, 2.13]	0.60
Hypertriglyceridemia	91/353	25.78%	53/178	29.78%	0.87 [0.65, 1.15]	0.32
Dysgeusia	8/34	23.53%	9/31	29.03%	0.81 [0.36, 1.84]	0.61
Rash	116/500	23.20%	53/256	20.70%	1.13 [0.85, 1.51]	0.41
Stomatitis	34/147	23.13%	9/78	11.54%	2.00 [1.02, 3.95]	0.04
Constipation	114/500	22.80%	49/256	19.14%	1.20 [0.89, 1.63]	0.23
Fatigue	33/147	22.45%	17/78	21.79%	1.02 [0.61, 1.72]	0.93
Decreased appetite	112/500	22.40%	43/256	16.80%	1.34 [0.97, 1.85]	0.08
Hypoesthesia	75/353	21.25%	24/178	13.48%	1.58 [1.03, 2.41]	0.04
Nail discoloration	7/34	20.59%	9/31	29.03%	0.71 [0.30, 1.67]	0.43
Paronychia	7/34	20.59%	0/31	0.00%	13.71 [0.82, 230.61]	0.07

AE, Adverse event; ALT, Alanine aminotransferase; AST, Aspartate aminotransferase; CI, Confidence interval; PD-1, Programmed death-1; PD-L1, Programmed death-ligand 1; PIC, PD-1/PD-L1 inhibitors plus chemotherapy; RR, Risk ratio; TRAE, Treatment-related adverse event.

**Table 4:** Grade 3-5 treatment-related adverse events (> 2% in the PIC group).

TRAEs	PIC		Chemotherapy		Risk ratio [95% CI]	P
	Event/total	%	Event/total	%		
Neutrophil count decreased	59/147	40.14%	28/78	35.90%	0.98 [0.70, 1.37]	0.89
White blood cell count decreased	40/147	27.21%	21/78	26.92%	0.86 [0.56, 1.32]	0.50
Leukopenia	89/353	25.21%	42/178	23.60%	1.07 [0.78, 1.47]	0.68
Neutropenia	115/500	23.00%	58/256	22.66%	0.98 [0.75, 1.29]	0.90
Decreased platelet count	12/113	10.62%	7/47	14.89%	0.71 [0.30, 1.70]	0.44
Anaemia	38/500	7.60%	13/256	5.08%	1.31 [0.73, 2.36]	0.37
AST increased	23/387	5.94%	4/209	1.91%	2.86 [1.02, 8.05]	0.05
ALT increased	21/387	5.43%	5/209	2.39%	2.11 [0.82, 5.47]	0.12
Hypoesthesia	16/353	4.53%	3/178	1.69%	2.69 [0.79, 9.11]	0.11
Asthenia	15/353	4.25%	4/178	2.25%	1.89 [0.64, 5.61]	0.25
Peripheral sensory neuropathy	18/500	3.60%	7/256	2.73%	1.21 [0.53, 2.80]	0.65
Lymphopenia	11/353	3.12%	6/178	3.37%	0.92 [0.35, 2.46]	0.87
Gamma-glutamyl transferase increased	10/353	2.83%	1/178	0.56%	5.04 [0.65, 39.08]	0.12
Hypokalemia	8/353	2.27%	4/178	2.25%	1.01 [0.31, 3.30]	0.99
Fatigue	3/147	2.04%	2/78	2.56%	0.62 [0.11, 3.61]	0.60

AE, Adverse event; ALT, Alanine aminotransferase; AST, Aspartate aminotransferase; CI, Confidence interval; PD-1, Programmed death-1; PD-L1, Programmed death-ligand 1; PIC, PD-1/PD-L1 inhibitors plus chemotherapy; RR, Risk ratio; TRAE, Treatment-related adverse event.

In the analysis of irAEs, the PIC group experienced higher rates of any grade hypothyroidism, and hyperthyroidism. All grade 3-5 irAEs were similar between the two groups (**Supplementary Table S6**, **S7**).

### Publication bias

**Figure 7:**
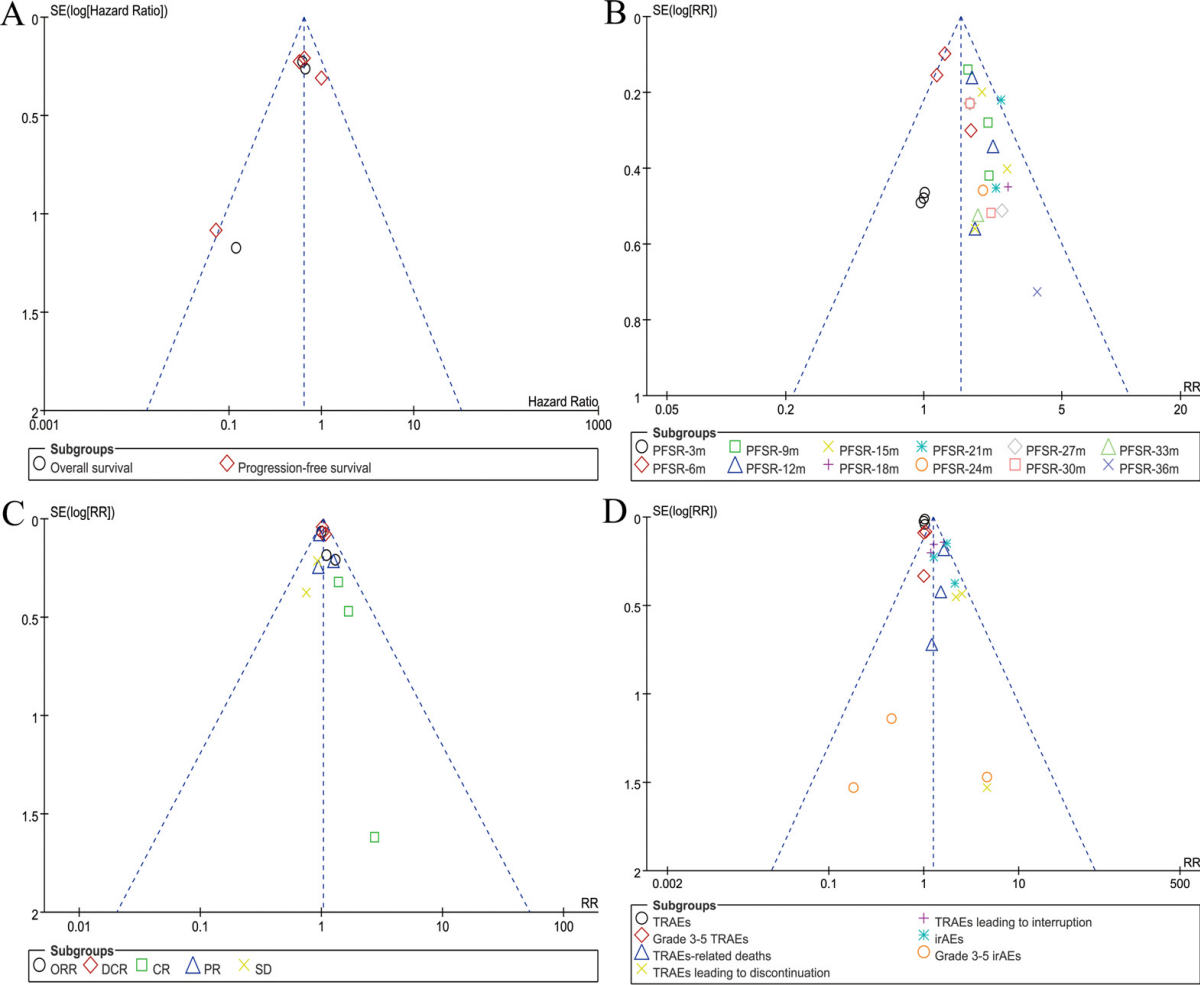
Funnel plots of survival (PD-L1-positive subgroup) **(A)**, PFSR **(B)**, responses **(C)**, and AEs summary **(D)**.

The funnel plot symmetry for survival (PD-L1-positive subgroup), PFSR, responses, and AEs summary indicated a low risk of publication bias (**Figure 7**).

## Discussion

Compared to other breast cancer subtypes, TNBC is associated with a higher likelihood of recurrence and metastasis, leading to a worse prognosis (2). Traditional treatment options for advanced TNBC have been limited, primarily relying on chemotherapy, which often yields suboptimal outcomes (3). In recent years, PD-1/PD-L1 inhibitors have been introduced as an important complement to chemotherapy and have shown promise in enhancing antitumor efficacy. However, the specific benefits and risks of this combination in Asian populations remain underexplored (9, 16). Our study is the first meta-analysis that aims to evaluate the efficacy and safety of PIC versus chemotherapy alone in Asian patients with advanced TNBC. The results showed that PIC therapy significantly improved survival, particularly in PD-L1-positive subgroup. However, the incidence of irAEs was increased in the PIC group. Both groups showed comparable response rates and TRAEs.

Our findings indicated a significant improvement in PFS in the PIC group. This result is consistent with the findings from pivotal studies such as IMpassion130, which demonstrated a significant improvement in PFS for patients with metastatic TNBC when treated with atezolizumab plus nab-paclitaxel (5). Likewise, the KEYNOTE-355 study showed similar results with pembrolizumab plus chemotherapy, particularly in PD-L1-positive subgroup (8). Interestingly, while we observed a trend toward improved OS in the PIC therapy group, statistical significance was not reached in the overall cohort. This observation mirrors the results from several other clinical trials, where improvements in OS have been difficult to demonstrate in early-phase trials of immune checkpoint inhibitors in TNBC (6, 7, 9). Several factors could account for this. First, the heterogeneous nature of TNBC means that patients respond differently to treatment, and the subgroup of patients benefiting from PIC therapy may be too small to show a significant OS benefit in the overall population (16). Second, the potential for crossover therapies-where patients in the chemotherapy group may receive subsequent immunotherapy upon progression-could dilute the observed OS differences (19). Furthermore, the duration of follow-up in many studies is often insufficient to fully capture the long-term survival effects of immunotherapy (7, 9). Immune checkpoint inhibitors tend to have a delayed onset of action, with benefits accruing over a longer period, which may be more apparent in studies with extended follow-up periods (20). Specifically, in the PD-L1-positive subgroup, PIC therapy significantly enhanced both PFS and OS. These findings align with the results from the IMpassion130 and KEYNOTE-355 studies, highlighting PD-L1 expression as an important biomarker for predicting the efficacy of PIC therapy in advanced TNBC (5, 8).

Although PFS was significantly improved with PIC therapy, our analysis found no significant difference in the ORR and DCR between the PIC and chemotherapy-alone groups. This finding, though unexpected, can be attributed to several factors that may influence how response rates are evaluated in immunotherapy-based treatments. PD-1/PD-L1 inhibitors primarily work by stimulating the body’s immune system to recognize and attack tumor cells, a process that can be slower and less apparent than the direct cytotoxic effects of chemotherapy (21). In contrast to traditional chemotherapy, which results in rapid tumor shrinkage, the effects of immunotherapy may take longer to manifest and can sometimes be delayed or not immediately reflected in conventional tumor response assessments, such as the standard RECIST criteria (22). For example, the phenomenon of “immune pseudoprogression”, where tumors temporarily increase in size due to immune cell infiltration, might lead to misleading conclusions regarding the effectiveness of treatment, as tumor growth during early treatment phases could be misinterpreted as a lack of response (23). In some cases, the tumor may stabilize or shrink later as the immune system mounts a sustained response, which cannot be captured through initial measurements of ORR. Furthermore, patients receiving PIC therapy may experience what is known as an “immune response flare”, where the immune system initially triggers inflammation in the tumor site, followed by subsequent tumor reduction (24). This complex immune response does not always translate into an immediate shrinkage of the tumor, further contributing to a lack of significant change in ORR. As observed in several clinical trials, this can explain why improvements in PFS, as seen in our study, might not be paralleled by increases in ORR and DCR, despite the potential for better long-term outcomes in terms of disease control (5–9). A more comprehensive understanding of how immune responses evolve over time, and how these dynamics differ from the cytotoxic response to chemotherapy, is critical in refining the criteria used to measure the effectiveness of combination therapies. Although our meta-analysis found no significant difference between the two groups in response rates, this does not necessarily diminish the potential of combining chemotherapy with PD-1/PD-L1 inhibitors. The complex and evolving nature of the immune response, coupled with the biological heterogeneity of TNBC, suggests that longer follow-up periods and more refined methods of assessing treatment response are needed.

In the safety assessment, we observed a higher incidence of irAEs in the PIC therapy group. The most frequently reported irAEs in this group included thyroid disorders (particularly hypothyroidism), dermatitis, infusion-related reactions, and gastrointestinal toxicities, such as diarrhea and colitis. Hypothyroidism is one of the most common irAEs associated with immune checkpoint inhibitors, resulting from autoimmune damage to the thyroid gland (25). Similarly, skin-related toxicities, including rash and pruritus, are also frequently observed. These side effects, while generally manageable, can significantly impact patients’ quality of life and may require dose adjustments, temporary treatment interruptions, or the use of corticosteroids (26). Our analysis also indicated an increased risk of grade 3-5 irAEs in the PIC therapy group, although the overall rate was still relatively low. The risk of severe irAEs is a well-known challenge with immunotherapy, particularly with agents that target the PD-1/PD-L1 axis (27). These toxicities can range from mild to life-threatening, and their occurrence often necessitates close monitoring and early intervention. In some cases, severe irAEs may lead to permanent discontinuation of the immune checkpoint inhibitor, although many of these AEs can be reversed with appropriate medical management, including the use of immune-suppressive agents like corticosteroids (28). In our study, the majority of AEs were reversible with prompt treatment, and the overall rate of treatment discontinuation due to toxicity was relatively low. However, the potential for these toxicities remains a significant concern when considering PIC therapy for advanced TNBC patients. Future studies should focus on optimizing treatment regimens to minimize toxicity, as well as identifying biomarkers that could predict which patients are most likely to experience severe irAEs.

This meta-analysis has several limitations. First, the included studies primarily enrolled patients from Western populations, with Asian patients constituting a smaller subset. This underrepresentation may limit the generalizability of our findings to the broader Asian TNBC population. Second, variations in PD-L1 assessment methods and cutoff values across studies could affect the comparability of results and the accurate identification of patients who would benefit from PIC therapy. Third, the relatively short follow-up durations in some trials may not adequately capture long-term survival benefits or late-onset AEs. Finally, potential publication bias and the exclusion of non-English studies may have influenced the comprehensiveness of our analysis.

## Conclusion

PIC significantly improves survival (OS and PFS) compared to chemotherapy alone in Asian patients with advanced TNBC, particularly in the PD-L1-positive subgroup. However, the increased incidence of irAEs necessitates careful patient selection and vigilant management. These findings support the incorporation of PD-1/PD-L1 inhibitors into treatment paradigms for this population, emphasizing the need for further research to optimize outcomes and minimize risks.

## Data Availability

The original contributions presented in the study are included in the article/**Supplementary Material**. Further inquiries can be directed to the corresponding author.

## Supplementary material

Supplementary Figure 1 Cochrane Risk Assessment.

Supplementary Figure 2 Forest plots of OSR at 3-36 months associated with PIC versus chemotherapy.

Supplementary Figure 3 Forest plots of PFSR at 3-36 months associated with PIC versus chemotherapy.

Supplementary Figure 4 Forest plots of AEs summary associated with PIC versus chemotherapy.
