# Supplementary figures and images for: PD-1/PD-L1 inhibitors plus chemotherapy versus chemotherapy alone for Asian patients with advanced triple-negative breast cancer: a phase III RCTs based meta-analysis
Source: Front Oncol. 2025 Feb 28;15:1540538. doi: 10.3389/fonc.2025.1540538 (PMC11906427; doi:10.3389/fonc.2025.1540538)

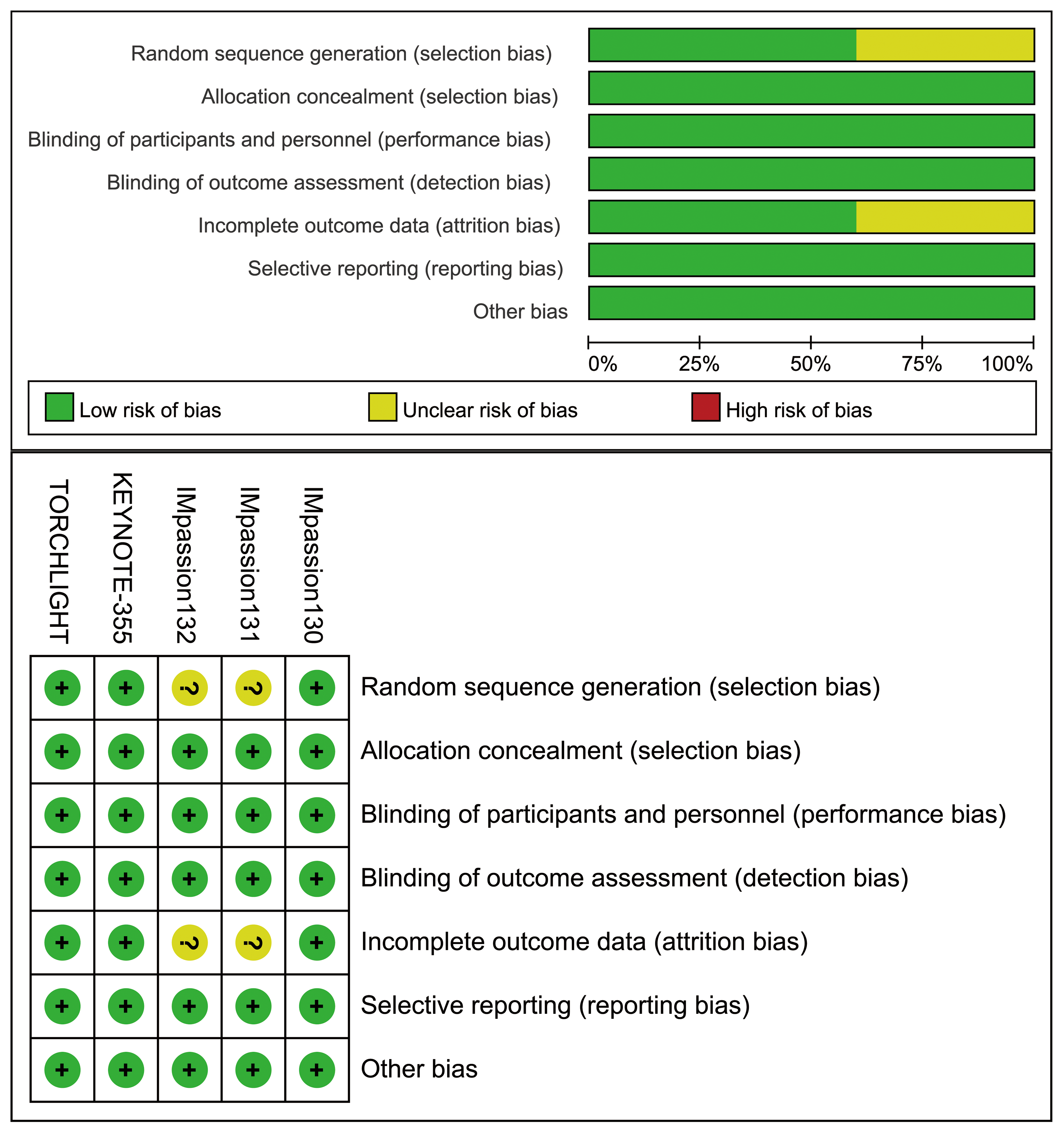

Supplement: Supplementary Figure 1 — Cochrane Risk Assessment. [file Image1.tif]

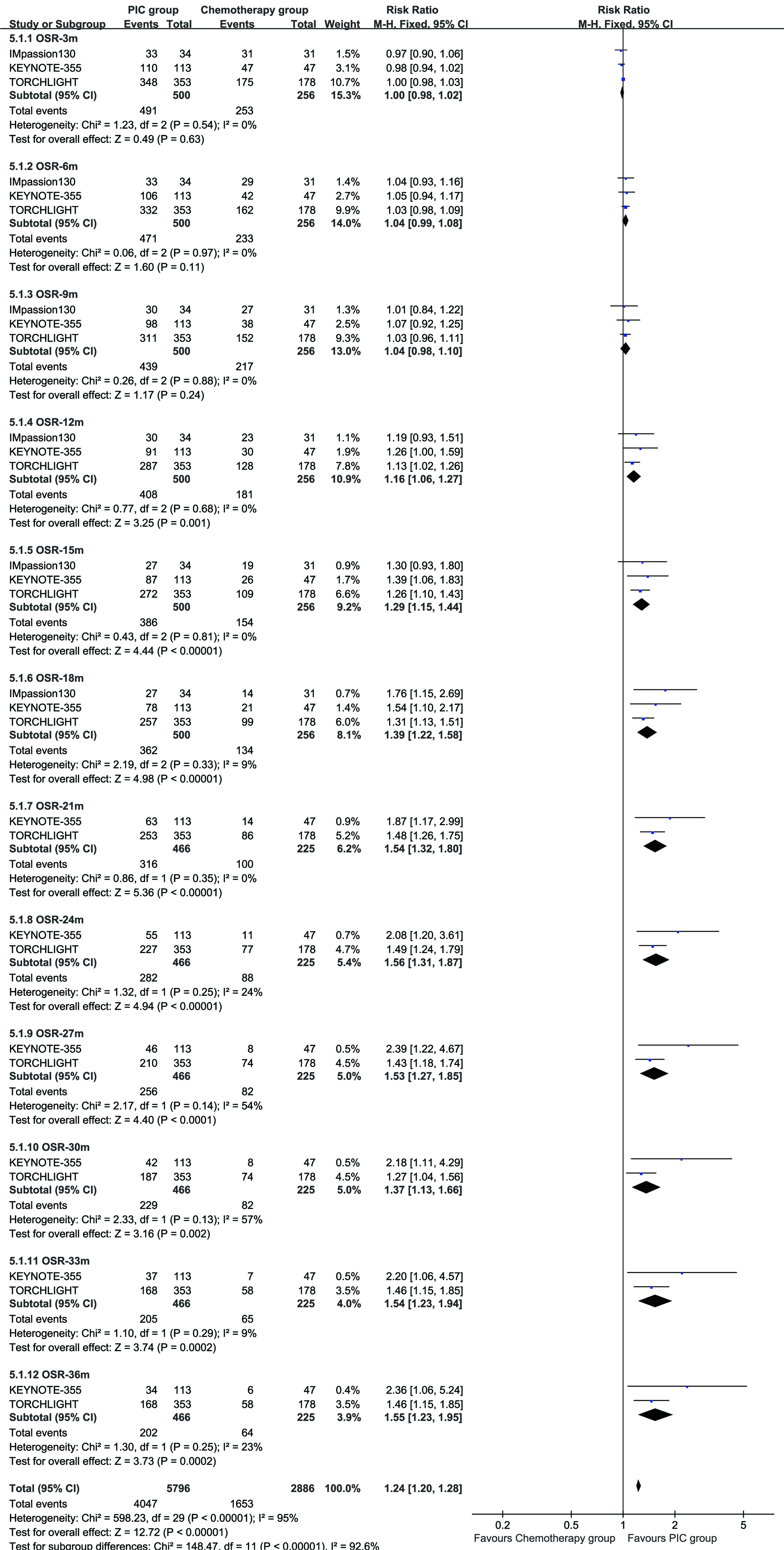

Supplement: Supplementary Figure 2 — Forest plots of OSR at 3-36 months associated with PIC versus chemotherapy. [file Image2.tif]

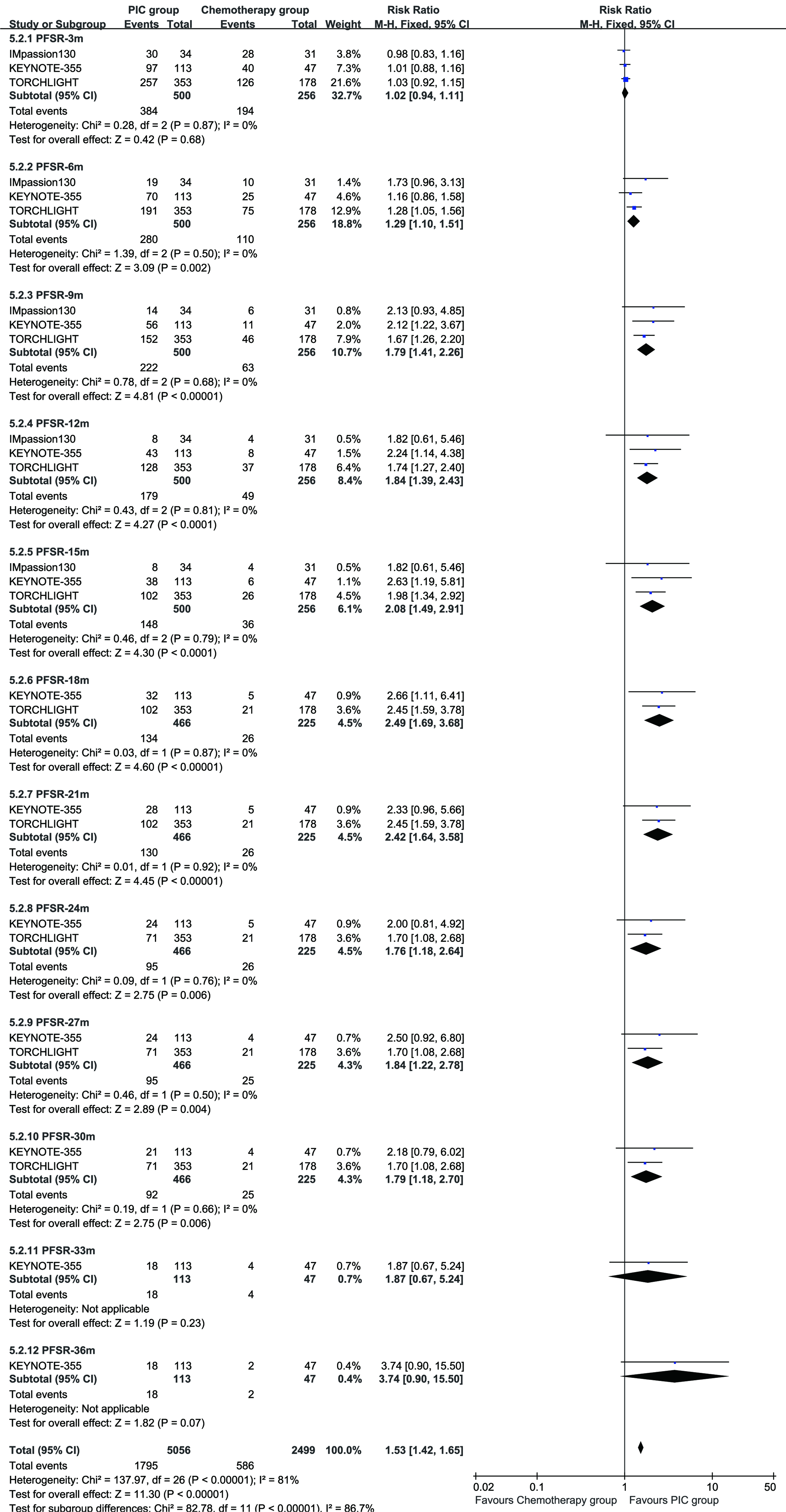

Supplement: Supplementary Figure 3 — Forest plots of PFSR at 3-36 months associated with PIC versus chemotherapy. [file Image3.tif]

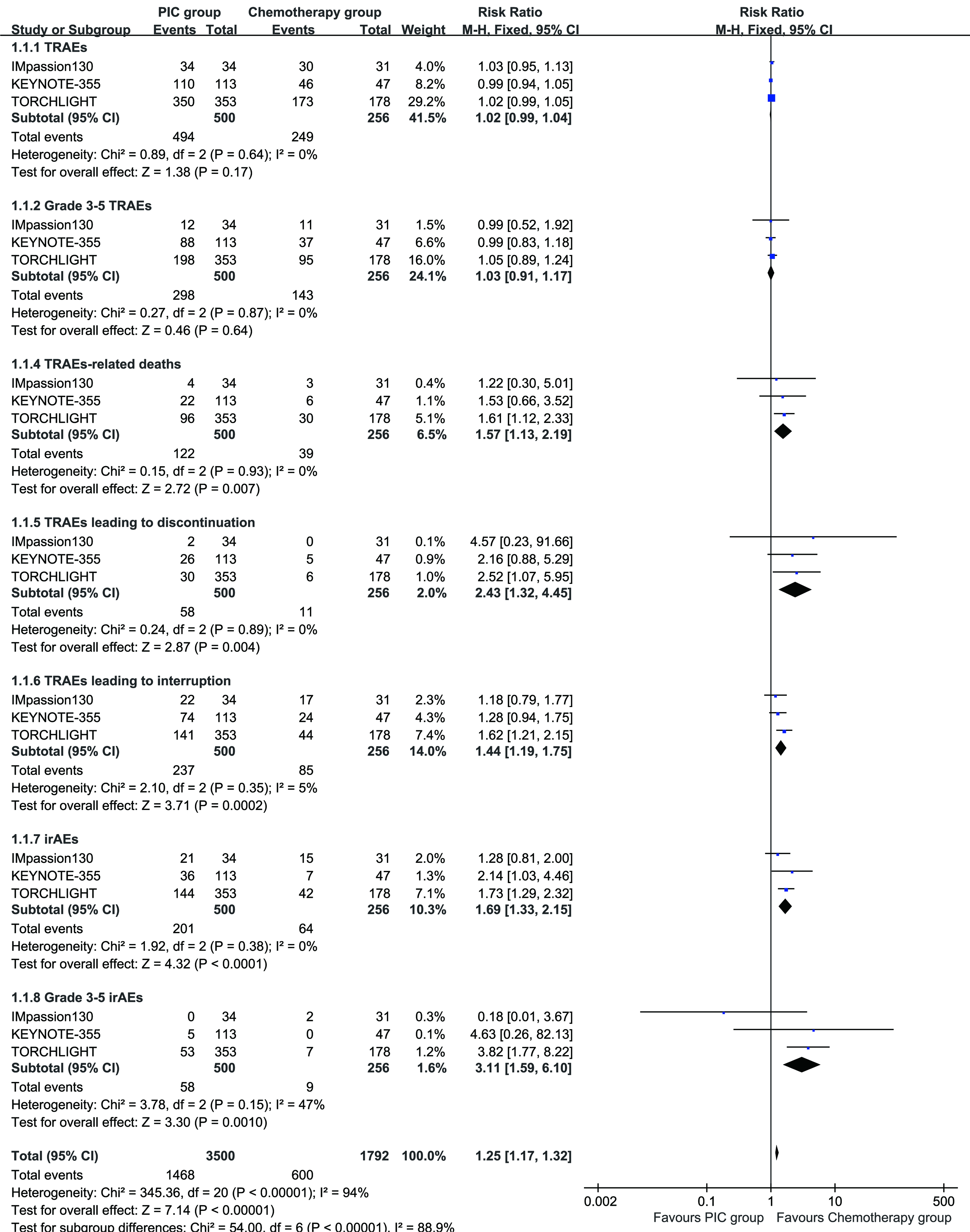

Supplement: Supplementary Figure 4 — Forest plots of AEs summary associated with PIC versus chemotherapy. [file Image4.tif]
