# Supplementary material for: PD-1/PD-L1 inhibitors plus chemotherapy versus chemotherapy alone for Asian patients with advanced triple-negative breast cancer: a phase III RCTs based meta-analysis
Source: Front Oncol. 2025 Feb 28;15:1540538. doi: 10.3389/fonc.2025.1540538 (PMC11906427; doi:10.3389/fonc.2025.1540538)
Supplement: Supplementary file 5 [file Table1.docx]

**Table S1** Search strategy.

| **PubMed**  The database was searched on November 12, 2024, n=133.  Search Strategy:  **#1 Search: Nivolumab[Title/Abstract] OR Pembrolizumab[Title/Abstract] OR Toripalimab[Title/Abstract] OR Sintilimab[Title/Abstract] OR Camrelizumab[Title/Abstract] OR Tislelizumab[Title/Abstract] OR Penpulimab[Title/Abstract] OR Zimberelimab[Title/Abstract] OR Serplulimab[Title/Abstract] OR Durvalumab[Title/Abstract] OR Atezolizumab[Title/Abstract] OR Envolizumab[Title/Abstract] OR Sugemalimab[Title/Abstract] OR Adebrelimab[Title/Abstract] OR Cemiplimab[Title/Abstract] OR Dostarlimab[Title/Abstract] OR Retifanlimab[Title/Abstract] OR Envafolimab[Title/Abstract] OR Suptavumab[Title/Abstract] OR Cadonilimab[Title/Abstract] OR Dostarlimab[Title/Abstract] OR Retifanlimab[Title/Abstract] Sort by: Most Recent n = 23387**  **#2 Search: Breast Cancer[Title/Abstract] OR Breast Neoplasm[Title/Abstract] OR Breast Tumor[Title/Abstract] Sort by: Most Recent n = 371411**  **#3 Search: Randomized[Title/Abstract] OR Randomly[Title/Abstract] OR Randomised[Title/Abstract] Sort by: Most Recent n = 1210056**  **#1 and #2 and #3 n = 133** |
| --- |
| **Web of Science**  The database was searched on November 12, 2024, n=1934.  Search Strategy:  Nivolumab OR Pembrolizumab OR Toripalimab OR Sintilimab OR Camrelizumab OR Tislelizumab OR Penpulimab OR Zimberelimab OR Serplulimab OR Durvalumab OR Atezolizumab OR Envolizumab OR Sugemalimab OR Adebrelimab OR Cemiplimab OR Dostarlimab OR Retifanlimab OR Envafolimab OR Suptavumab OR Cadonilimab OR Dostarlimab OR Retifanlimab (Abstract) AND Breast Cancer OR Breast Neoplasm OR Breast Tumor (Abstract) AND Randomized OR Randomly OR Randomised (Abstract) and Preprint Citation Index (Exclude - Database) |
| **EMBASE**  The database was searched on November 12, 2024, n=276.  Search Strategy:  (Nivolumab:ti,ab,kw OR Pembrolizumab:ti,ab,kw OR Toripalimab:ti,ab,kw OR Sintilimab:ti,ab,kw OR Camrelizumab:ti,ab,kw OR Tislelizumab:ti,ab,kw OR Penpulimab:ti,ab,kw OR Zimberelimab:ti,ab,kw OR Serplulimab:ti,ab,kw OR Durvalumab:ti,ab,kw OR Atezolizumab:ti,ab,kw OR Envolizumab:ti,ab,kw OR Sugemalimab:ti,ab,kw OR Adebrelimab:ti,ab,kw OR Cemiplimab:ti,ab,kw OR Envafolimab:ti,ab,kw OR Suptavumab:ti,ab,kw OR Cadonilimab:ti,ab,kw OR Dostarlimab:ti,ab,kw OR Retifanlimab:ti,ab,kw) AND (Breast Cancer:ti,ab,kw OR Breast Neoplasm:ti,ab,kw OR Breast Tumor:ti,ab,kw) AND **(Randomly**:ti,ab,kw **OR Randomised**:ti,ab,kw **OR**  **Randomized** :ti,ab,kw**)** |
| **Cochrane Library**  The database was searched on November 12, 2024, n=42.  Search Strategy:  (Nivolumab OR Pembrolizumab OR Toripalimab OR Sintilimab OR Camrelizumab OR Tislelizumab OR Penpulimab OR Zimberelimab OR Serplulimab OR Durvalumab OR Atezolizumab OR Envolizumab OR Sugemalimab OR Adebrelimab OR Cemiplimab OR Dostarlimab OR Retifanlimab OR Envafolimab OR Suptavumab OR Cadonilimab**)** in Title Abstract Keyword AND (Breast Cancer OR Breast Neoplasm OR Breast Tumor**)** in Title Abstract Keyword AND (**Randomized OR Randomly OR Randomised)** in Title Abstract Keyword - (Word variations have been searched) |
| **ScienceDirect**  The database was searched on November 12, 2024, n=766.  Search Strategy:  Title, abstract, keywords: ((“Nivolumab” OR “Pembrolizumab” OR “Toripalimab” OR “Sintilimab” OR “Camrelizumab” OR “Tislelizumab” OR “Penpulimab” OR “Zimberelimab” OR “Serplulimab” OR “Durvalumab” OR “Atezolizumab” OR “Envolizumab” OR “Sugemalimab” OR “Adebrelimab” OR “Cemiplimab” OR “Dostarlimab” OR “Retifanlimab” OR “Envafolimab” OR “Suptavumab” OR “Cadonilimab”) AND (“Breast Cancer” OR “Breast Neoplasm” OR “Breast Tumor”) AND (“**Randomized**” **OR Randomly**” **OR** “**Randomised**”)) |
| **Scopus**  The database was searched on November 12, 2024, n=259.  Search Strategy:  (TITLE-ABS-KEY (Nivolumab OR Pembrolizumab OR Toripalimab OR Sintilimab OR Camrelizumab OR Tislelizumab OR Penpulimab OR Zimberelimab OR Serplulimab OR Durvalumab OR Atezolizumab OR Envolizumab OR Sugemalimab OR Adebrelimab OR Cemiplimab OR Dostarlimab OR Retifanlimab OR Envafolimab OR Suptavumab OR Cadonilimab OR Dostarlimab OR Retifanlimab) AND TITLE-ABS-KEY (Breast Cancer OR Breast Neoplasm OR Breast Tumor) AND TITLE-ABS-KEY (Randomized OR Randomly OR Randomised) ) |

**Note:** The combined text and medical subject heading (MeSH) terms used were: “**PD-1/PD-L1 inhibitors**”, “Breast Cancer”, and “**Randomized**”.
