# Supplementary material for: PD-1/PD-L1 inhibitors plus chemotherapy versus chemotherapy alone for Asian patients with advanced triple-negative breast cancer: a phase III RCTs based meta-analysis
Source: Front Oncol. 2025 Feb 28;15:1540538. doi: 10.3389/fonc.2025.1540538 (PMC11906427; doi:10.3389/fonc.2025.1540538)
Supplement: Supplementary file 6 [file Table2.doc]

**Table S2 Methodological quality assessments (Jadad scale) of the included studies.**

| **Study** | | **Randomization** | **Masking** | **Accountability of all patients** | **Quality (score)** |
| --- | --- | --- | --- | --- | --- |
| IMpassion130 (NCT02425891) | Emens 2021[5], Iwata 2019[16], Schmid 2018[17] | ** | ** | * | 5 |
| IMpassion131 (NCT03125902) | Miles 2021[6] | ** | ** | * | 5 |
| IMpassion132 (NCT03371017) | Dent 2024[7] | ** | ** | * | 5 |
| KEYNOTE-355 (NCT02819518) | Im 2024[8], Hattori[18] | ** | ** | * | 5 |
| TORCHLIGHT (NCT03777579) | Jiang 2024[9] | ** | ** | * | 5 |
