# Supplementary material for: PD-1/PD-L1 inhibitors plus chemotherapy versus chemotherapy alone for Asian patients with advanced triple-negative breast cancer: a phase III RCTs based meta-analysis
Source: Front Oncol. 2025 Feb 28;15:1540538. doi: 10.3389/fonc.2025.1540538 (PMC11906427; doi:10.3389/fonc.2025.1540538)
Supplement: Supplementary file 7 [file Table3.doc]

**Table S3** GRADE quality assessment by therapeutic strategy and study design for the outcomes.

| **Primary outcomes** | **No. of Participants** | | **Differences (95%CI) ^a^** | **Quality Assessment** | | | | | **Quality** |
| --- | --- | --- | --- | --- | --- | --- | --- | --- | --- |
|  | **PIC** | **Chemotherapy** |  | **Risk of Bias^b^** | **Inconsistency** | **Indirectness** | **Imprecision** | **Publication Bias^c^** |  |
| **Survival** |  |  |  |  |  |  |  |  |  |
| OS | 599 | 349 | 0.78 [0.55, 1.12] | Low | Serious (-1) | No indirectness | No imprecision | Unlikely | Medium |
| PFS | 656 | 379 | 0.74 [0.62, 0.88] | Low | No inconsistency | No indirectness | No imprecision | Unlikely | High |
| **Survival for PD-L1-positive patients** |  |  |  |  |  |  |  |  |  |
| OS | 313 | 149 | 0.62 [0.44, 0.86] | Low | No inconsistency | No indirectness | No imprecision | Unlikely | High |
| PFS | 370 | 179 | 0.66 [0.50, 0.86] | Low | No inconsistency | No indirectness | No imprecision | Unlikely | High |
| **Survival rate** |  |  |  |  |  |  |  |  |  |
| **OSR** |  |  |  |  |  |  |  |  |  |
| OSR-3m | 491/500 | 253/256 | 1.00 [0.98, 1.02] | Low | No inconsistency | No indirectness | No imprecision | Unlikely | High |
| OSR-6m | 471/500 | 233/256 | 1.04 [0.99, 1.08] | Low | No inconsistency | No indirectness | No imprecision | Unlikely | High |
| OSR-9m | 439/500 | 217/256 | 1.04 [0.98, 1.10] | Low | No inconsistency | No indirectness | No imprecision | Unlikely | High |
| OSR-12m | 408/500 | 181/256 | 1.16 [1.06, 1.27] | Low | No inconsistency | No indirectness | No imprecision | Unlikely | High |
| OSR-15m | 386/500 | 154/256 | 1.29 [1.15, 1.44] | Low | No inconsistency | No indirectness | No imprecision | Unlikely | High |
| OSR-18m | 362/500 | 134/256 | 1.39 [1.22, 1.58] | Low | No inconsistency | No indirectness | No imprecision | Unlikely | High |
| OSR-21m | 316/466 | 100/225 | 1.54 [1.32, 1.80] | Low | No inconsistency | No indirectness | No imprecision | Unlikely | High |
| OSR-24m | 282/466 | 88/225 | 1.56 [1.31, 1.87] | Low | No inconsistency | No indirectness | No imprecision | Unlikely | High |
| OSR-27m | 256/466 | 82/225 | 1.53 [1.27, 1.85] | Low | No inconsistency | No indirectness | No imprecision | Unlikely | High |
| OSR-30m | 229/466 | 82/225 | 1.37 [1.13, 1.66] | Low | No inconsistency | No indirectness | No imprecision | Unlikely | High |
| OSR-33m | 205/466 | 65/225 | 1.54 [1.23, 1.94] | Low | No inconsistency | No indirectness | No imprecision | Unlikely | High |
| OSR-36m | 202/466 | 64/225 | 1.55 [1.23, 1.95] | Low | No inconsistency | No indirectness | No imprecision | Unlikely | High |
| **PFSR** |  |  |  |  |  |  |  |  |  |
| PFSR-3m | 384/500 | 194/256 | 1.02 [0.94, 1.11] | Low | No inconsistency | No indirectness | No imprecision | Unlikely | High |
| PFSR-6m | 280/500 | 110/256 | 1.29 [1.10, 1.51] | Low | No inconsistency | No indirectness | No imprecision | Unlikely | High |
| PFSR-9m | 222/500 | 63/256 | 1.79 [1.41, 2.26] | Low | No inconsistency | No indirectness | No imprecision | Unlikely | High |
| PFSR-12m | 179/500 | 49/256 | 1.84 [1.39, 2.43] | Low | No inconsistency | No indirectness | No imprecision | Unlikely | High |
| PFSR-15m | 148/500 | 36/256 | 2.08 [1.49, 2.91] | Low | No inconsistency | No indirectness | No imprecision | Unlikely | High |
| PFSR-18m | 134/466 | 26/225 | 2.49 [1.69, 3.68] | Low | No inconsistency | No indirectness | No imprecision | Unlikely | High |
| PFSR-21m | 130/466 | 26/225 | 2.42 [1.64, 3.58] | Low | No inconsistency | No indirectness | No imprecision | Unlikely | High |
| PFSR-24m | 95/466 | 26/225 | 1.76 [1.18, 2.64] | Low | No inconsistency | No indirectness | No imprecision | Unlikely | High |
| PFSR-27m | 95/466 | 25/225 | 1.84 [1.22, 2.78] | Low | No inconsistency | No indirectness | No imprecision | Unlikely | High |
| PFSR-30m | 92/466 | 25/225 | 1.79 [1.18, 2.70] | Low | No inconsistency | No indirectness | No imprecision | Unlikely | High |
| PFSR-33m | 18/113 | 4/47 | 1.87 [0.67, 5.24] | Low | No inconsistency | No indirectness | No imprecision | Unlikely | High |
| PFSR-36m | 18/113 | 2/47 | 3.74 [0.90, 15.50] | Low | No inconsistency | No indirectness | No imprecision | Unlikely | High |
| **Responses** |  |  |  |  |  |  |  |  |  |
| ORR | 306/500 | 151/256 | 1.05 [0.93, 1.18] | Low | No inconsistency | No indirectness | No imprecision | Unlikely | High |
| DCR | 425/500 | 212/256 | 1.03 [0.96, 1.10] | Low | No inconsistency | No indirectness | No imprecision | Unlikely | High |
| CR | 54/500 | 17/256 | 1.50 [0.90, 2.51] | Low | No inconsistency | No indirectness | No imprecision | Unlikely | High |
| PR | 252/500 | 134/256 | 0.98 [0.85, 1.14] | Low | No inconsistency | No indirectness | No imprecision | Unlikely | High |
| SD | 51/147 | 30/78 | 0.87 [0.60, 1.25] | Low | No inconsistency | No indirectness | No imprecision | Unlikely | High |
| **Safety summary** |  |  |  |  |  |  |  |  |  |
| TRAEs | 494/500 | 249/256 | 1.02 [0.99, 1.04] | Low | No inconsistency | No indirectness | No imprecision | Unlikely | High |
| Grade 3-5 TRAEs | 298/500 | 143/256 | 1.03 [0.91, 1.17] | Low | No inconsistency | No indirectness | No imprecision | Unlikely | High |
| TRAEs-related deaths | 122/500 | 39/256 | 1.57 [1.13, 2.19] | Low | No inconsistency | No indirectness | No imprecision | Unlikely | High |
| TRAEs leading to discontinuation | 58/500 | 11/256 | 2.43 [1.32, 4.45] | Low | No inconsistency | No indirectness | No imprecision | Unlikely | High |
| TRAEs leading to interruption | 237/500 | 85/256 | 1.44 [1.19, 1.75] | Low | No inconsistency | No indirectness | No imprecision | Unlikely | High |
| irAEs | 201/500 | 64/256 | 1.69 [1.33, 2.15] | Low | No inconsistency | No indirectness | No imprecision | Unlikely | High |
| Grade 3-5 irAEs | 58/500 | 9/256 | 3.11 [1.59, 6.10] | Low | No inconsistency | No indirectness | No imprecision | Unlikely | High |
| **TRAEs** |  |  |  |  |  |  |  |  |  |
| **Any grade** |  |  |  |  |  |  |  |  |  |
| Leukopenia | 257/353 | 134/178 | 0.97 [0.87, 1.07] | Low | No inconsistency | No indirectness | No imprecision | Unlikely | High |
| Alopecia | 315/500 | 154/256 | 1.08 [0.96, 1.22] | Low | No inconsistency | No indirectness | No imprecision | Unlikely | High |
| Anaemia | 280/500 | 132/256 | 1.05 [0.92, 1.21] | Low | No inconsistency | No indirectness | No imprecision | Unlikely | High |
| Neutrophil count decreased | 80/147 | 39/78 | 1.02 [0.79, 1.32] | Low | No inconsistency | No indirectness | No imprecision | Unlikely | High |
| Neutropenia | 266/500 | 123/256 | 1.08 [0.95, 1.23] | Low | No inconsistency | No indirectness | No imprecision | Unlikely | High |
| White blood cell count decreased | 67/147 | 30/78 | 1.44 [0.41, 5.02] | Low | Serious (-1) | No indirectness | No imprecision | Unlikely | Medium |
| AST increased | 165/387 | 92/209 | 0.92 [0.77, 1.11] | Low | No inconsistency | No indirectness | No imprecision | Unlikely | High |
| ALT increased | 163/387 | 93/209 | 0.91 [0.75, 1.10] | Low | No inconsistency | No indirectness | No imprecision | Unlikely | High |
| Nasopharyngitis | 11/34 | 3/31 | 3.34 [1.03, 10.88] | Low | No inconsistency | No indirectness | No imprecision | Unlikely | High |
| Nausea | 161/500 | 60/256 | 1.39 [1.08, 1.78] | Low | No inconsistency | No indirectness | No imprecision | Unlikely | High |
| Asthenia | 111/353 | 39/178 | 1.44 [1.05, 1.97] | Low | No inconsistency | No indirectness | No imprecision | Unlikely | High |
| Decreased platelet count | 31/113 | 11/47 | 1.17 [0.64, 2.13] | Low | No inconsistency | No indirectness | No imprecision | Unlikely | High |
| Hypertriglyceridemia | 91/353 | 53/178 | 0.87 [0.65, 1.15] | Low | No inconsistency | No indirectness | No imprecision | Unlikely | High |
| Dysgeusia | 8/34 | 9/31 | 0.81 [0.36, 1.84] | Low | No inconsistency | No indirectness | No imprecision | Unlikely | High |
| Rash | 116/500 | 53/256 | 1.13 [0.85, 1.51] | Low | No inconsistency | No indirectness | No imprecision | Unlikely | High |
| Stomatitis | 34/147 | 9/78 | 2.00 [1.02, 3.95] | Low | No inconsistency | No indirectness | No imprecision | Unlikely | High |
| Constipation | 114/500 | 49/256 | 1.20 [0.89, 1.63] | Low | No inconsistency | No indirectness | No imprecision | Unlikely | High |
| Fatigue | 33/147 | 17/78 | 1.02 [0.61, 1.72] | Low | No inconsistency | No indirectness | No imprecision | Unlikely | High |
| Decreased appetite | 112/500 | 43/256 | 1.34 [0.97, 1.85] | Low | No inconsistency | No indirectness | No imprecision | Unlikely | High |
| Hypoesthesia | 75/353 | 24/178 | 1.58 [1.03, 2.41] | Low | No inconsistency | No indirectness | No imprecision | Unlikely | High |
| Nail discoloration | 7/34 | 9/31 | 0.71 [0.30, 1.67] | Low | No inconsistency | No indirectness | No imprecision | Unlikely | High |
| Paronychia | 7/34 | 0/31 | 13.71 [0.82, 230.61] | Low | No inconsistency | No indirectness | No imprecision | Unlikely | High |
| Vomiting | 96/500 | 34/256 | 1.43 [1.00, 2.05] | Low | No inconsistency | No indirectness | No imprecision | Unlikely | High |
| Malaise | 27/147 | 13/78 | 0.98 [0.55, 1.75] | Low | No inconsistency | No indirectness | No imprecision | Unlikely | High |
| Hypothyroidism | 71/387 | 14/209 | 2.57 [1.51, 4.39] | Low | No inconsistency | No indirectness | No imprecision | Unlikely | High |
| Peripheral sensory neuropathy | 89/500 | 47/256 | 1.08 [0.79, 1.47] | Low | No inconsistency | No indirectness | No imprecision | Unlikely | High |
| Myalgia | 6/34 | 7/31 | 0.78 [0.29, 2.07] | Low | No inconsistency | No indirectness | No imprecision | Unlikely | High |
| Dry skin | 6/34 | 6/31 | 0.91 [0.33, 2.53] | Low | No inconsistency | No indirectness | No imprecision | Unlikely | High |
| Headache | 6/34 | 4/31 | 1.37 [0.43, 4.40] | Low | No inconsistency | No indirectness | No imprecision | Unlikely | High |
| Pruritis | 6/34 | 2/31 | 2.74 [0.60, 12.56] | Low | No inconsistency | No indirectness | No imprecision | Unlikely | High |
| Pyrexia | 68/387 | 29/209 | 1.30 [0.87, 1.94] | Low | No inconsistency | No indirectness | No imprecision | Unlikely | High |
| Gamma-glutamyl transferase increased | 55/353 | 21/178 | 1.32 [0.83, 2.11] | Low | No inconsistency | No indirectness | No imprecision | Unlikely | High |
| Urinary tract infection | 60/387 | 29/209 | 1.08 [0.72, 1.62] | Low | No inconsistency | No indirectness | No imprecision | Unlikely | High |
| Peripheral edema | 59/387 | 46/209 | 0.70 [0.50, 0.99] | Low | No inconsistency | No indirectness | No imprecision | Unlikely | High |
| Hyperuricemia | 52/353 | 27/178 | 0.97 [0.63, 1.49] | Low | No inconsistency | No indirectness | No imprecision | Unlikely | High |
| Hypoalbuminemia | 52/353 | 25/178 | 1.05 [0.67, 1.63] | Low | No inconsistency | No indirectness | No imprecision | Unlikely | High |
| Diarrhea | 56/387 | 27/209 | 1.14 [0.74, 1.75] | Low | No inconsistency | No indirectness | No imprecision | Unlikely | High |
| Hypokalemia | 49/353 | 16/178 | 1.54 [0.90, 2.64] | Low | No inconsistency | No indirectness | No imprecision | Unlikely | High |
| Blood bilirubin increased | 47/353 | 23/178 | 1.03 [0.65, 1.64] | Low | No inconsistency | No indirectness | No imprecision | Unlikely | High |
| Lymphopenia | 46/353 | 27/178 | 0.86 [0.55, 1.33] | Low | No inconsistency | No indirectness | No imprecision | Unlikely | High |
| Blood alkaline phosphatase increased | 46/353 | 19/178 | 1.22 [0.74, 2.02] | Low | No inconsistency | No indirectness | No imprecision | Unlikely | High |
| Hyperglycemia | 45/353 | 28/178 | 0.81 [0.52, 1.25] | Low | No inconsistency | No indirectness | No imprecision | Unlikely | High |
| Hypercholesterolemia | 44/353 | 30/178 | 0.74 [0.48, 1.13] | Low | No inconsistency | No indirectness | No imprecision | Unlikely | High |
| Arthralgia | 4/34 | 4/31 | 0.91 [0.25, 3.34] | Low | No inconsistency | No indirectness | No imprecision | Unlikely | High |
| Weight decreased | 40/353 | 16/178 | 1.26 [0.73, 2.19] | Low | No inconsistency | No indirectness | No imprecision | Unlikely | High |
| Blood lactate dehydrogenase increased | 38/353 | 16/178 | 1.20 [0.69, 2.09] | Low | No inconsistency | No indirectness | No imprecision | Unlikely | High |
| Hyponatremia | 36/353 | 19/178 | 0.96 [0.56, 1.62] | Low | No inconsistency | No indirectness | No imprecision | Unlikely | High |
| Thrombocytopenia | 35/353 | 19/178 | 0.93 [0.55, 1.58] | Low | No inconsistency | No indirectness | No imprecision | Unlikely | High |
| Cough | 38/387 | 24/209 | 0.85 [0.52, 1.38] | Low | No inconsistency | No indirectness | No imprecision | Unlikely | High |
| Insomnia | 35/387 | 19/209 | 0.98 [0.58, 1.67] | Low | No inconsistency | No indirectness | No imprecision | Unlikely | High |
| Oedema | 3/34 | 3/31 | 0.91 [0.20, 4.19] | Low | No inconsistency | No indirectness | No imprecision | Unlikely | High |
| Pharyngitis | 2/34 | 3/31 | 0.61 [0.11, 3.40] | Low | No inconsistency | No indirectness | No imprecision | Unlikely | High |
| **Grade 3-5** |  |  |  |  |  |  |  |  |  |
| Neutrophil count decreased | 59/147 | 28/78 | 0.98 [0.70, 1.37] | Low | No inconsistency | No indirectness | No imprecision | Unlikely | High |
| White blood cell count decreased | 40/147 | 21/78 | 0.86 [0.56, 1.32] | Low | No inconsistency | No indirectness | No imprecision | Unlikely | High |
| Leukopenia | 89/353 | 42/178 | 1.07 [0.78, 1.47] | Low | No inconsistency | No indirectness | No imprecision | Unlikely | High |
| Neutropenia | 115/500 | 58/256 | 0.98 [0.75, 1.29] | Low | No inconsistency | No indirectness | No imprecision | Unlikely | High |
| Decreased platelet count | 12/113 | 7/47 | 0.71 [0.30, 1.70] | Low | No inconsistency | No indirectness | No imprecision | Unlikely | High |
| Anaemia | 38/500 | 13/256 | 1.31 [0.73, 2.36] | Low | No inconsistency | No indirectness | No imprecision | Unlikely | High |
| AST increased | 23/387 | 4/209 | 2.86 [1.02, 8.05] | Low | No inconsistency | No indirectness | No imprecision | Unlikely | High |
| ALT increased | 21/387 | 5/209 | 2.11 [0.82, 5.47] | Low | No inconsistency | No indirectness | No imprecision | Unlikely | High |
| Hypoesthesia | 16/353 | 3/178 | 2.69 [0.79, 9.11] | Low | No inconsistency | No indirectness | No imprecision | Unlikely | High |
| Asthenia | 15/353 | 4/178 | 1.89 [0.64, 5.61] | Low | No inconsistency | No indirectness | No imprecision | Unlikely | High |
| Peripheral sensory neuropathy | 18/500 | 7/256 | 1.21 [0.53, 2.80] | Low | No inconsistency | No indirectness | No imprecision | Unlikely | High |
| Lymphopenia | 11/353 | 6/178 | 0.92 [0.35, 2.46] | Low | No inconsistency | No indirectness | No imprecision | Unlikely | High |
| Gamma-glutamyl transferase increased | 10/353 | 1/178 | 5.04 [0.65, 39.08] | Low | No inconsistency | No indirectness | No imprecision | Unlikely | High |
| Hypokalemia | 8/353 | 4/178 | 1.01 [0.31, 3.30] | Low | No inconsistency | No indirectness | No imprecision | Unlikely | High |
| Fatigue | 3/147 | 2/78 | 0.62 [0.11, 3.61] | Low | No inconsistency | No indirectness | No imprecision | Unlikely | High |
| Hypertriglyceridemia | 6/353 | 2/178 | 1.51 [0.31, 7.42] | Low | No inconsistency | No indirectness | No imprecision | Unlikely | High |
| Rash | 8/500 | 0/256 | 4.33 [0.56, 33.23] | Low | No inconsistency | No indirectness | No imprecision | Unlikely | High |
| Nausea | 7/500 | 3/256 | 1.14 [0.31, 4.28] | Low | No inconsistency | No indirectness | No imprecision | Unlikely | High |
| Vomiting | 7/500 | 1/256 | 2.13 [0.46, 9.83] | Low | No inconsistency | No indirectness | No imprecision | Unlikely | High |
| Stomatitis | 2/147 | 0/78 | 2.11 [0.10, 43.04] | Low | No inconsistency | No indirectness | No imprecision | Unlikely | High |
| Malaise | 2/147 | 0/78 | 2.11 [0.10, 43.04] | Low | No inconsistency | No indirectness | No imprecision | Unlikely | High |
| Diarrhea | 4/387 | 1/209 | 2.02 [0.23, 17.91] | Low | No inconsistency | No indirectness | No imprecision | Unlikely | High |
| Urinary tract infection | 4/387 | 1/209 | 2.02 [0.23, 17.91] | Low | No inconsistency | No indirectness | No imprecision | Unlikely | High |
| Decreased appetite | 5/500 | 4/256 | 0.65 [0.18, 2.34] | Low | No inconsistency | No indirectness | No imprecision | Unlikely | High |
| Weight decreased | 3/353 | 0/178 | 3.54 [0.18, 68.15] | Low | No inconsistency | No indirectness | No imprecision | Unlikely | High |
| Thrombocytopenia | 2/353 | 0/178 | 2.53 [0.12, 52.38] | Low | No inconsistency | No indirectness | No imprecision | Unlikely | High |
| Hyponatremia | 2/353 | 1/178 | 1.01 [0.09, 11.05] | Low | No inconsistency | No indirectness | No imprecision | Unlikely | High |
| Blood alkaline phosphatase increased | 1/353 | 1/178 | 0.50 [0.03, 8.01] | Low | No inconsistency | No indirectness | No imprecision | Unlikely | High |
| Blood lactate dehydrogenase increased | 1/353 | 0/178 | 1.52 [0.06, 37.05] | Low | No inconsistency | No indirectness | No imprecision | Unlikely | High |
| Hyperglycemia | 1/353 | 0/178 | 1.52 [0.06, 37.05] | Low | No inconsistency | No indirectness | No imprecision | Unlikely | High |
| Hypercholesterolemia | 1/353 | 0/178 | 1.52 [0.06, 37.05] | Low | No inconsistency | No indirectness | No imprecision | Unlikely | High |
| Peripheral edema | 1/387 | 1/209 | 0.50 [0.03, 8.01] | Low | No inconsistency | No indirectness | No imprecision | Unlikely | High |
| Insomnia | 1/387 | 0/209 | 1.52 [0.06, 37.05] | Low | No inconsistency | No indirectness | No imprecision | Unlikely | High |
| Constipation | 1/500 | 1/256 | 0.45 [0.06, 3.35] | Low | No inconsistency | No indirectness | No imprecision | Unlikely | High |
| Myalgia | 0/34 | 1/31 | 0.30 [0.01, 7.22] | Low | No inconsistency | No indirectness | No imprecision | Unlikely | High |
| Blood bilirubin increased | 0/353 | 2/178 | 0.10 [0.00, 2.10] | Low | No inconsistency | No indirectness | No imprecision | Unlikely | High |
| Hyperuricemia | 0/353 | 1/178 | 0.17 [0.01, 4.12] | Low | No inconsistency | No indirectness | No imprecision | Unlikely | High |
| **irAEs** |  |  |  |  |  |  |  |  |  |
| **Any grade** |  |  |  |  |  |  |  |  |  |
| Hypothyroidism | 72/500 | 8/256 | 4.41 [2.19, 8.88] | Low | No inconsistency | No indirectness | No imprecision | Unlikely | High |
| Dermatitis | 50/500 | 24/256 | 1.26 [0.81, 1.96] | Low | No inconsistency | No indirectness | No imprecision | Unlikely | High |
| Infusion reactions | 10/113 | 2/47 | 2.08 [0.47, 9.13] | Low | No inconsistency | No indirectness | No imprecision | Unlikely | High |
| Hyperthyroidism | 32/500 | 3/256 | 4.81 [1.61, 14.32] | Low | No inconsistency | No indirectness | No imprecision | Unlikely | High |
| Pneumonitis | 13/500 | 0/256 | 5.27 [0.96, 28.90] | Low | No inconsistency | No indirectness | No imprecision | Unlikely | High |
| Hepatitis | 10/500 | 9/256 | 0.80 [0.34, 1.89] | Low | No inconsistency | No indirectness | No imprecision | Unlikely | High |
| Adrenal insufficiency | 7/466 | 0/225 | 3.61 [0.45, 28.88] | Low | No inconsistency | No indirectness | No imprecision | Unlikely | High |
| Thyroiditis | 6/466 | 0/225 | 3.23 [0.40, 26.02] | Low | No inconsistency | No indirectness | No imprecision | Unlikely | High |
| Vasculitis | 1/113 | 1/47 | 0.42 [0.03, 6.51] | Low | No inconsistency | No indirectness | No imprecision | Unlikely | High |
| Myositis | 0/34 | 1/31 | 0.30 [0.01, 7.22] | Low | No inconsistency | No indirectness | No imprecision | Unlikely | High |
| Colitis | 0/113 | 1/47 | 0.14 [0.01, 3.38] | Low | No inconsistency | No indirectness | No imprecision | Unlikely | High |
| **Grade 3-5** |  |  |  |  |  |  |  |  |  |
| Dermatitis | 8/500 | 0/256 | 3.83 [0.48, 30.63] | Low | No inconsistency | No indirectness | No imprecision | Unlikely | High |
| Infusion reactions | 1/113 | 0/47 | 1.26 [0.05, 30.46] | Low | No inconsistency | No indirectness | No imprecision | Unlikely | High |
| Adrenal insufficiency | 3/466 | 0/225 | 1.80 [0.20, 16.18] | Low | No inconsistency | No indirectness | No imprecision | Unlikely | High |
| Pneumonitis | 3/500 | 0/256 | 1.82 [0.20, 16.32] | Low | No inconsistency | No indirectness | No imprecision | Unlikely | High |
| Hepatitis | 1/500 | 0/256 | 1.26 [0.05, 30.46] | Low | No inconsistency | No indirectness | No imprecision | Unlikely | High |

**Abbreviations:** AE: Adverse event; ALT: Alanine aminotransferase; AST: Aspartate aminotransferase; CR: Complete response; DCR: Disease control rate; HR: Hazard ratio; irAE: Immune-related adverse event; ORR: Objective response rate; OS: Overall survival; OSR: Overall survival rate; PD-1: Programmed death-1; PD-L1: Programmed death-ligand 1; PFS: Progression-free survival; PFSR: Progression-free survival rate; PIC: PD-1/PD-L1 inhibitors plus chemotherapy; PR: Partial response; RCT: Randomized controlled trial; RR: Risk ratio; SD: Stable disease; TNBC: Triple-negative breast cancer; TRAE: Treatment-related adverse event.

^a^ Differences: HR for OS and PFS; RR for OSR, PFSR, responses, and AEs.

^b^ Risk of bias assessed using the Jadad scale for randomized controlled trials.

^c^ Publication bias was explored through visual inspection of the funnel plots.
