# Supplementary material for: PD-1/PD-L1 inhibitors plus chemotherapy versus chemotherapy alone for Asian patients with advanced triple-negative breast cancer: a phase III RCTs based meta-analysis
Source: Front Oncol. 2025 Feb 28;15:1540538. doi: 10.3389/fonc.2025.1540538 (PMC11906427; doi:10.3389/fonc.2025.1540538)
Supplement: Supplementary file 8 [file Table4.doc]

**Table S4** Any grade treatment-related adverse events (All).

| **TRAEs** | **PIC** | |  | **Chemotherapy** | | **Risk ratio [95% CI]** | **P** |
| --- | --- | --- | --- | --- | --- | --- | --- |
|  | **Event/total** | **%** |  | **Event/total** | **%** |  |  |
| Leukopenia | 257/353 | 72.80% |  | 134/178 | 75.28% | 0.97 [0.87, 1.07] | 0.53 |
| Alopecia | 315/500 | 63.00% |  | 154/256 | 60.16% | 1.08 [0.96, 1.22] | 0.18 |
| Anaemia | 280/500 | 56.00% |  | 132/256 | 51.56% | 1.05 [0.92, 1.21] | 0.48 |
| Neutrophil count decreased | 80/147 | 54.42% |  | 39/78 | 50.00% | 1.02 [0.79, 1.32] | 0.88 |
| Neutropenia | 266/500 | 53.20% |  | 123/256 | 48.05% | 1.08 [0.95, 1.23] | 0.26 |
| White blood cell count decreased | 67/147 | 45.58% |  | 30/78 | 38.46% | 1.44 [0.41, 5.02] | 0.57 |
| AST increased | 165/387 | 42.64% |  | 92/209 | 44.02% | 0.92 [0.77, 1.11] | 0.41 |
| ALT increased | 163/387 | 42.12% |  | 93/209 | 44.50% | 0.91 [0.75, 1.10] | 0.34 |
| Nasopharyngitis | 11/34 | 32.35% |  | 3/31 | 9.68% | 3.34 [1.03, 10.88] | 0.05 |
| Nausea | 161/500 | 32.20% |  | 60/256 | 23.44% | 1.39 [1.08, 1.78] | 0.01 |
| Asthenia | 111/353 | 31.44% |  | 39/178 | 21.91% | 1.44 [1.05, 1.97] | 0.03 |
| Decreased platelet count | 31/113 | 27.43% |  | 11/47 | 23.40% | 1.17 [0.64, 2.13] | 0.60 |
| Hypertriglyceridemia | 91/353 | 25.78% |  | 53/178 | 29.78% | 0.87 [0.65, 1.15] | 0.32 |
| Dysgeusia | 8/34 | 23.53% |  | 9/31 | 29.03% | 0.81 [0.36, 1.84] | 0.61 |
| Rash | 116/500 | 23.20% |  | 53/256 | 20.70% | 1.13 [0.85, 1.51] | 0.41 |
| Stomatitis | 34/147 | 23.13% |  | 9/78 | 11.54% | 2.00 [1.02, 3.95] | 0.04 |
| Constipation | 114/500 | 22.80% |  | 49/256 | 19.14% | 1.20 [0.89, 1.63] | 0.23 |
| Fatigue | 33/147 | 22.45% |  | 17/78 | 21.79% | 1.02 [0.61, 1.72] | 0.93 |
| Decreased appetite | 112/500 | 22.40% |  | 43/256 | 16.80% | 1.34 [0.97, 1.85] | 0.08 |
| Hypoesthesia | 75/353 | 21.25% |  | 24/178 | 13.48% | 1.58 [1.03, 2.41] | 0.04 |
| Nail discoloration | 7/34 | 20.59% |  | 9/31 | 29.03% | 0.71 [0.30, 1.67] | 0.43 |
| Paronychia | 7/34 | 20.59% |  | 0/31 | 0.00% | 13.71 [0.82, 230.61] | 0.07 |
| Vomiting | 96/500 | 19.20% |  | 34/256 | 13.28% | 1.43 [1.00, 2.05] | 0.05 |
| Malaise | 27/147 | 18.37% |  | 13/78 | 16.67% | 0.98 [0.55, 1.75] | 0.95 |
| Hypothyroidism | 71/387 | 18.35% |  | 14/209 | 6.70% | 2.57 [1.51, 4.39] | 0.0005 |
| Peripheral sensory neuropathy | 89/500 | 17.80% |  | 47/256 | 18.36% | 1.08 [0.79, 1.47] | 0.63 |
| Myalgia | 6/34 | 17.65% |  | 7/31 | 22.58% | 0.78 [0.29, 2.07] | 0.62 |
| Dry skin | 6/34 | 17.65% |  | 6/31 | 19.35% | 0.91 [0.33, 2.53] | 0.86 |
| Headache | 6/34 | 17.65% |  | 4/31 | 12.90% | 1.37 [0.43, 4.40] | 0.60 |
| Pruritis | 6/34 | 17.65% |  | 2/31 | 6.45% | 2.74 [0.60, 12.56] | 0.20 |
| Pyrexia | 68/387 | 17.57% |  | 29/209 | 13.88% | 1.30 [0.87, 1.94] | 0.20 |
| Gamma-glutamyl transferase increased | 55/353 | 15.58% |  | 21/178 | 11.80% | 1.32 [0.83, 2.11] | 0.25 |
| Urinary tract infection | 60/387 | 15.50% |  | 29/209 | 13.88% | 1.08 [0.72, 1.62] | 0.71 |
| Peripheral edema | 59/387 | 15.25% |  | 46/209 | 22.01% | 0.70 [0.50, 0.99] | 0.05 |
| Hyperuricemia | 52/353 | 14.73% |  | 27/178 | 15.17% | 0.97 [0.63, 1.49] | 0.89 |
| Hypoalbuminemia | 52/353 | 14.73% |  | 25/178 | 14.04% | 1.05 [0.67, 1.63] | 0.83 |
| Diarrhea | 56/387 | 14.47% |  | 27/209 | 12.92% | 1.14 [0.74, 1.75] | 0.55 |
| Hypokalemia | 49/353 | 13.88% |  | 16/178 | 8.99% | 1.54 [0.90, 2.64] | 0.11 |
| Blood bilirubin increased | 47/353 | 13.31% |  | 23/178 | 12.92% | 1.03 [0.65, 1.64] | 0.90 |
| Lymphopenia | 46/353 | 13.03% |  | 27/178 | 15.17% | 0.86 [0.55, 1.33] | 0.50 |
| Blood alkaline phosphatase increased | 46/353 | 13.03% |  | 19/178 | 10.67% | 1.22 [0.74, 2.02] | 0.44 |
| Hyperglycemia | 45/353 | 12.75% |  | 28/178 | 15.73% | 0.81 [0.52, 1.25] | 0.34 |
| Hypercholesterolemia | 44/353 | 12.46% |  | 30/178 | 16.85% | 0.74 [0.48, 1.13] | 0.17 |
| Arthralgia | 4/34 | 11.76% |  | 4/31 | 12.90% | 0.91 [0.25, 3.34] | 0.89 |
| Weight decreased | 40/353 | 11.33% |  | 16/178 | 8.99% | 1.26 [0.73, 2.19] | 0.41 |
| Blood lactate dehydrogenase increased | 38/353 | 10.76% |  | 16/178 | 8.99% | 1.20 [0.69, 2.09] | 0.52 |
| Hyponatremia | 36/353 | 10.20% |  | 19/178 | 10.67% | 0.96 [0.56, 1.62] | 0.87 |
| Thrombocytopenia | 35/353 | 9.92% |  | 19/178 | 10.67% | 0.93 [0.55, 1.58] | 0.78 |
| Cough | 38/387 | 9.82% |  | 24/209 | 11.48% | 0.85 [0.52, 1.38] | 0.50 |
| Insomnia | 35/387 | 9.04% |  | 19/209 | 9.09% | 0.98 [0.58, 1.67] | 0.95 |
| Oedema | 3/34 | 8.82% |  | 3/31 | 9.68% | 0.91 [0.20, 4.19] | 0.91 |
| Pharyngitis | 2/34 | 5.88% |  | 3/31 | 9.68% | 0.61 [0.11, 3.40] | 0.57 |

**Abbreviations:** AE: Adverse event; ALT: Alanine aminotransferase; AST: Aspartate aminotransferase; CI: Confidence interval; PD-1: Programmed death-1; PD-L1: Programmed death-ligand 1; PIC: PD-1/PD-L1 inhibitors plus chemotherapy; RR: Risk ratio; TRAE: Treatment-related adverse event.
