# Supplementary material for: PD-1/PD-L1 inhibitors plus chemotherapy versus chemotherapy alone for Asian patients with advanced triple-negative breast cancer: a phase III RCTs based meta-analysis
Source: Front Oncol. 2025 Feb 28;15:1540538. doi: 10.3389/fonc.2025.1540538 (PMC11906427; doi:10.3389/fonc.2025.1540538)
Supplement: Supplementary file 9 [file Table5.doc]

**Table S5** Grade 3-5 treatment-related adverse events (All)).

| **TRAEs** | **PIC** | |  | **Chemotherapy** | | **Risk ratio [95% CI]** | **P** |
| --- | --- | --- | --- | --- | --- | --- | --- |
|  | **Event/total** | **%** |  | **Event/total** | **%** |  |  |
| Neutrophil count decreased | 59/147 | 40.14% |  | 28/78 | 35.90% | 0.98 [0.70, 1.37] | 0.89 |
| White blood cell count decreased | 40/147 | 27.21% |  | 21/78 | 26.92% | 0.86 [0.56, 1.32] | 0.50 |
| Leukopenia | 89/353 | 25.21% |  | 42/178 | 23.60% | 1.07 [0.78, 1.47] | 0.68 |
| Neutropenia | 115/500 | 23.00% |  | 58/256 | 22.66% | 0.98 [0.75, 1.29] | 0.90 |
| Decreased platelet count | 12/113 | 10.62% |  | 7/47 | 14.89% | 0.71 [0.30, 1.70] | 0.44 |
| Anaemia | 38/500 | 7.60% |  | 13/256 | 5.08% | 1.31 [0.73, 2.36] | 0.37 |
| AST increased | 23/387 | 5.94% |  | 4/209 | 1.91% | 2.86 [1.02, 8.05] | 0.05 |
| ALT increased | 21/387 | 5.43% |  | 5/209 | 2.39% | 2.11 [0.82, 5.47] | 0.12 |
| Hypoesthesia | 16/353 | 4.53% |  | 3/178 | 1.69% | 2.69 [0.79, 9.11] | 0.11 |
| Asthenia | 15/353 | 4.25% |  | 4/178 | 2.25% | 1.89 [0.64, 5.61] | 0.25 |
| Peripheral sensory neuropathy | 18/500 | 3.60% |  | 7/256 | 2.73% | 1.21 [0.53, 2.80] | 0.65 |
| Lymphopenia | 11/353 | 3.12% |  | 6/178 | 3.37% | 0.92 [0.35, 2.46] | 0.87 |
| Gamma-glutamyl transferase increased | 10/353 | 2.83% |  | 1/178 | 0.56% | 5.04 [0.65, 39.08] | 0.12 |
| Hypokalemia | 8/353 | 2.27% |  | 4/178 | 2.25% | 1.01 [0.31, 3.30] | 0.99 |
| Fatigue | 3/147 | 2.04% |  | 2/78 | 2.56% | 0.62 [0.11, 3.61] | 0.60 |
| Hypertriglyceridemia | 6/353 | 1.70% |  | 2/178 | 1.12% | 1.51 [0.31, 7.42] | 0.61 |
| Rash | 8/500 | 1.60% |  | 0/256 | 0.00% | 4.33 [0.56, 33.23] | 0.16 |
| Nausea | 7/500 | 1.40% |  | 3/256 | 1.17% | 1.14 [0.31, 4.28] | 0.84 |
| Vomiting | 7/500 | 1.40% |  | 1/256 | 0.39% | 2.13 [0.46, 9.83] | 0.33 |
| Stomatitis | 2/147 | 1.36% |  | 0/78 | 0.00% | 2.11 [0.10, 43.04] | 0.63 |
| Malaise | 2/147 | 1.36% |  | 0/78 | 0.00% | 2.11 [0.10, 43.04] | 0.63 |
| Diarrhea | 4/387 | 1.03% |  | 1/209 | 0.48% | 2.02 [0.23, 17.91] | 0.53 |
| Urinary tract infection | 4/387 | 1.03% |  | 1/209 | 0.48% | 2.02 [0.23, 17.91] | 0.53 |
| Decreased appetite | 5/500 | 1.00% |  | 4/256 | 1.56% | 0.65 [0.18, 2.34] | 0.51 |
| Weight decreased | 3/353 | 0.85% |  | 0/178 | 0.00% | 3.54 [0.18, 68.15] | 0.40 |
| Thrombocytopenia | 2/353 | 0.57% |  | 0/178 | 0.00% | 2.53 [0.12, 52.38] | 0.55 |
| Hyponatremia | 2/353 | 0.57% |  | 1/178 | 0.56% | 1.01 [0.09, 11.05] | 0.99 |
| Blood alkaline phosphatase increased | 1/353 | 0.28% |  | 1/178 | 0.56% | 0.50 [0.03, 8.01] | 0.63 |
| Blood lactate dehydrogenase increased | 1/353 | 0.28% |  | 0/178 | 0.00% | 1.52 [0.06, 37.05] | 0.80 |
| Hyperglycemia | 1/353 | 0.28% |  | 0/178 | 0.00% | 1.52 [0.06, 37.05] | 0.80 |
| Hypercholesterolemia | 1/353 | 0.28% |  | 0/178 | 0.00% | 1.52 [0.06, 37.05] | 0.80 |
| Peripheral edema | 1/387 | 0.26% |  | 1/209 | 0.48% | 0.50 [0.03, 8.01] | 0.63 |
| Insomnia | 1/387 | 0.26% |  | 0/209 | 0.00% | 1.52 [0.06, 37.05] | 0.80 |
| Constipation | 1/500 | 0.20% |  | 1/256 | 0.39% | 0.45 [0.06, 3.35] | 0.44 |
| Myalgia | 0/34 | 0.00% |  | 1/31 | 3.23% | 0.30 [0.01, 7.22] | 0.46 |
| Blood bilirubin increased | 0/353 | 0.00% |  | 2/178 | 1.12% | 0.10 [0.00, 2.10] | 0.14 |
| Hyperuricemia | 0/353 | 0.00% |  | 1/178 | 0.56% | 0.17 [0.01, 4.12] | 0.27 |

**Abbreviations:** AE: Adverse event; ALT: Alanine aminotransferase; AST: Aspartate aminotransferase; CI: Confidence interval; PD-1: Programmed death-1; PD-L1: Programmed death-ligand 1; PIC: PD-1/PD-L1 inhibitors plus chemotherapy; RR: Risk ratio; TRAE: Treatment-related adverse event.
