# Supplementary material for: PD-1/PD-L1 inhibitors plus chemotherapy versus chemotherapy alone for Asian patients with advanced triple-negative breast cancer: a phase III RCTs based meta-analysis
Source: Front Oncol. 2025 Feb 28;15:1540538. doi: 10.3389/fonc.2025.1540538 (PMC11906427; doi:10.3389/fonc.2025.1540538)
Supplement: Supplementary file 10 [file Table6.doc]

**Table S6** Any grade immune-related adverse events.

| **irAEs** | **PIC** | |  | **Chemotherapy** | | **Risk ratio [95% CI]** | **P** |
| --- | --- | --- | --- | --- | --- | --- | --- |
|  | **Event/total** | **%** |  | **Event/total** | **%** |  |  |
| Hypothyroidism | 72/500 | 14.40% |  | 8/256 | 3.13% | 4.41 [2.19, 8.88] | < 0.0001 |
| Dermatitis | 50/500 | 10.00% |  | 24/256 | 9.38% | 1.26 [0.81, 1.96] | 0.30 |
| Infusion reactions | 10/113 | 8.85% |  | 2/47 | 4.26% | 2.08 [0.47, 9.13] | 0.33 |
| Hyperthyroidism | 32/500 | 6.40% |  | 3/256 | 1.17% | 4.81 [1.61, 14.32] | 0.005 |
| Pneumonitis | 13/500 | 2.60% |  | 0/256 | 0.00% | 5.27 [0.96, 28.90] | 0.06 |
| Hepatitis | 10/500 | 2.00% |  | 9/256 | 3.52% | 0.80 [0.34, 1.89] | 0.61 |
| Adrenal insufficiency | 7/466 | 1.50% |  | 0/225 | 0.00% | 3.61 [0.45, 28.88] | 0.23 |
| Thyroiditis | 6/466 | 1.29% |  | 0/225 | 0.00% | 3.23 [0.40, 26.02] | 0.27 |
| Vasculitis | 1/113 | 0.88% |  | 1/47 | 2.13% | 0.42 [0.03, 6.51] | 0.53 |
| Myositis | 0/34 | 0.00% |  | 1/31 | 3.23% | 0.30 [0.01, 7.22] | 0.46 |
| Colitis | 0/113 | 0.00% |  | 1/47 | 2.13% | 0.14 [0.01, 3.38] | 0.23 |

**Abbreviations:** AE: Adverse event; CI: Confidence interval; irAE: Immune-related adverse event; PD-1: Programmed death-1; PD-L1: Programmed death-ligand 1; PIC: PD-1/PD-L1 inhibitors plus chemotherapy; RR: Risk ratio.
