# Supplementary material for: PD-1/PD-L1 inhibitors plus chemotherapy versus chemotherapy alone for Asian patients with advanced triple-negative breast cancer: a phase III RCTs based meta-analysis
Source: Front Oncol. 2025 Feb 28;15:1540538. doi: 10.3389/fonc.2025.1540538 (PMC11906427; doi:10.3389/fonc.2025.1540538)
Supplement: Supplementary file 11 [file Table7.doc]

**Table S7** Grade 3-5 immune-related adverse events.

| **irAEs** | **PIC** | |  | **Chemotherapy** | | **Risk ratio [95% CI]** | **P** |
| --- | --- | --- | --- | --- | --- | --- | --- |
|  | **Event/total** | **%** |  | **Event/total** | **%** |  |  |
| Dermatitis | 8/500 | 1.60% |  | 0/256 | 0.00% | 3.83 [0.48, 30.63] | 0.21 |
| Infusion reactions | 1/113 | 0.88% |  | 0/47 | 0.00% | 1.26 [0.05, 30.46] | 0.89 |
| Adrenal insufficiency | 3/466 | 0.64% |  | 0/225 | 0.00% | 1.80 [0.20, 16.18] | 0.60 |
| Pneumonitis | 3/500 | 0.60% |  | 0/256 | 0.00% | 1.82 [0.20, 16.32] | 0.59 |
| Hepatitis | 1/500 | 0.20% |  | 0/256 | 0.00% | 1.26 [0.05, 30.46] | 0.89 |

**Abbreviations:** AE: Adverse event; CI: Confidence interval; irAE: Immune-related adverse event; PD-1: Programmed death-1; PD-L1: Programmed death-ligand 1; PIC: PD-1/PD-L1 inhibitors plus chemotherapy; RR: Risk ratio.
